# PvGTSeq and PvCRiSP: Two amplicon-based targeted sequencing panels for *Plasmodium vivax*

**DOI:** 10.1371/journal.pntd.0013663

**Published:** 2026-05-14

**Authors:** Paulo C. Manrique-Valverde, Chloe M. Hasund, Katrina A. Kelley, Jorge-Eduardo Amaya-Romero, Myriam Arévalo-Herrera, Raphael Brosula, Jose E. Calzada, Stella M. Chenet, Vladimir Corredor, Angela M. Early, Gustavo Fontecha, David A. Forero-Peña, Sócrates Herrera, Justin T. Lana, Margaret Laws, Reza Niles-Robin, Nicanor Obaldia, Ana M. Santamaria, Philipp Schwabl, Sarah Auburn, Daniel E. Neafsey

**Affiliations:** 1 Department of Immunology and Infectious Diseases, Harvard T.H. Chan School of Public Health, Boston, Massachusetts, United States of America; 2 Infectious Disease and Microbiome Program, Broad Institute, Cambridge, Massachusetts, United States of America; 3 Malaria Vaccine and Drug Development Center, Cali, Colombia; 4 Caucaseco Scientific Research Center, Cali, Colombia; 5 Universidad de Panamá, Panamá, República de Panamá; 6 Departamento de Investigación en Parasitología, Instituto Conmemorativo Gorgas de Estudios de la Salud (ICGES), Panamá, República de Panamá; 7 Instituto de Investigación de Enfermedades Tropicales (IET), Universidad Nacional Toribio Rodríguez de Mendoza de Amazonas (UNTRM), Chachapoyas, Peru; 8 Facultad de Medicina (FAMED), Universidad Nacional Toribio Rodríguez de Mendoza de Amazonas (UNTRM), Chachapoyas, Peru; 9 Departamento de Salud Pública, Facultad de Medicina, Universidad Nacional de Colombia, Bogotá, Colombia; 10 Instituto de Investigaciones en Microbiología, Facultad de Ciencias, Universidad Nacional Autónoma de Honduras, Tegucigalpa, Honduras; 11 Biomedical Research and Therapeutic Vaccines Institute, Ciudad Bolívar, Venezuela; 12 Clinton Health Access Initiative, Boston, Massachusetts, United States of America; 13 National Malaria Program, Ministry of Health, Georgetown, Guyana; 14 Institute of International Animal Health/One Health, Friedrich-Loeffler-Institut, Greifswald (Insel Riems), Germany; 15 Global and Tropical Health Division, Menzies School of Health Research and Charles Darwin University, Darwin, Australia; 16 Centre for Tropical Medicine and Global Health, Nuffield Department of Medicine, University of Oxford, Oxford, United Kingdom; 17 Mahidol-Oxford Tropical Medicine Research Unit, Mahidol University, Bangkok, Thailand‌‌; Ohio State University, UNITED STATES OF AMERICA

## Abstract

*Plasmodium vivax* is the main cause of malaria outside of sub-Saharan Africa, and in many settings it presents significant challenges to malaria elimination efforts. Despite some control successes in the Americas, regional annual case counts of malaria have increased by over 25% between 2014 and 2023, largely driven by *P. vivax.* Genomic surveillance can play a key role in understanding the extent to which disease persistence represents indigenous transmission as opposed to introduction of new strains through migration, and whether specific variants evade control measures. Efforts to make *P. vivax* genomic surveillance more cost-effective have led to the development of targeted sequencing-based methods, which strike a varying balance between assay sensitivity and breadth/informativeness. We introduce two new highly sensitive multiplexed amplicon sequencing panels for *P. vivax*: PvGTSeq and PvCRiSP. PvGTSeq requires selective whole-genome amplification (sWGA) and contains 249 amplicons—36 for antimalarial resistance and 213 for population structure—optimized for Latin America but applicable to all continents. PvCRiSP features four highly polymorphic amplicons that operate without sWGA and is designed to estimate complexity of infection (COI), identify instances of clonal transmission, and characterize recurrent episodes. Both panels use a single multiplex PCR with non-proprietary reagents, achieve ≥75% amplicon recovery at parasitemias as low as five parasites/μL, and PvCRiSP remains effective with low quality DNA. PvGTSeq showed high sequencing accuracy (error rate 3.85e-4% - 2.87e-3%), and both panels efficiently detected alleles from minority clones in simulated polyclonal infections. We validated both panels with samples from Colombia, Guyana, Honduras, Panama, and Venezuela, and performed in-silico assessments using data from 16 countries worldwide, confirming that these two panels have high power to discriminate samples and assign global geographic origin to imported cases. These panels will therefore be useful tools for *P. vivax* molecular surveillance in diverse geographic settings.

## Author summary

Genomic surveillance of malaria parasites has proven highly valuable for tracking the spatial and temporal distribution of drug resistance markers, measuring population connectivity, and differentiating locally transmitted vs. imported infections, among other use cases. We have designed and validated two Illumina-based multiplexed PCR amplicon sequencing panels for *P. vivax*: PvGTSeq and PvCRiSP. The PvGTSeq panel incorporates 249 amplicons, covering candidate genes associated with drug resistance as well as amplicons capturing polymorphic regions particularly useful for fine scale spatial resolution of parasite populations in Latin America. PvCRiSP comprises four highly diverse and ultra-sensitive amplicons, it is intended to estimate complexity of infection (COI), detect clonal transmission events, and analyze recurrent infection episodes without a costly and effort-intensive pre-amplification step. Both panels reliably generate genotypic data for patient samples with parasitemia levels as low as five parasites/μL and do not use proprietary reagents. These panels are suited for use in diverse geographic regions, with particular utility for studying *P. vivax* in the Americas—a region that is facing significant challenges in malaria control and elimination.

## Introduction

*Plasmodium vivax* is the main cause of malaria outside of Africa. Each year, 6–7 million cases are reported, and in the Americas this Plasmodium species causes 76% of all malaria cases [1]. Despite efforts to reduce malaria, *P. vivax* possesses biological characteristics that make it resilient to current control measures. Compared to *Plasmodium falciparum*, *P. vivax* has an earlier developmental commitment to transmissible sexual stages [2] and can persist as a dormant hypnozoite in the liver that can reactivate weeks or months after the primary infection [3]. In addition to these biological advantages, several factors complicate the control and elimination of this parasite in the Americas: the high frequency of submicroscopic and asymptomatic infections [4], its prevalence in remote rural and border regions with limited health surveillance [5], and inadequate patient compliance with radical cure treatment (7–14 days primaquine) to clear hypnozoites from the liver [6].

Despite successful malaria control and elimination efforts in some countries across the Americas, there has been an increase in *P. vivax* cases since 2014—even in areas that previously reported fewer than 1,000 cases annually or where *P. falciparum* was the dominant parasite [1]. Although Venezuela has experienced the most dramatic increase in cases [7], other countries such as Colombia, Costa Rica, Guyana, Ecuador, Guatemala, Honduras, Nicaragua, and Panama have also shown increases [1]. The drivers of *P. vivax* persistence and resurgence are complex, and control programs therefore require cost-effective tools to monitor these population changes and enable evidence-based decision-making.

Genomic surveillance can play an important role in this goal, and the development of targeted sequencing-based methods for *P. falciparum* has made this tool more cost-effective and easy to transfer to National Malaria Control Programs [8–12]. Recently, the use of genomic surveillance has expanded to *P. vivax* with a variety of use cases [13–20]. The most frequent use case has been characterizing population connectivity and distinguishing imported infections from those that are locally acquired. Other applications include characterizing candidate antimalarial resistance-related genes and potential blood-stage vaccine targets, as well as identifying amplicons to distinguish between infection relapses, recrudescences, and reinfections.

Here, we introduce two new amplicon panels for targeted Illumina sequencing of *P. vivax*. The first, PvGTSeq, is a multipurpose panel composed of 249 amplicons, including 213 highly heterozygous amplicons for assessing parasite diversity, relatedness, and population structure at three geographic scales: global, intra-continental, and sub-national in Latin America and the Caribbean. This panel has enhanced sensitivity for identifying imported infections in the Americas, while maintaining utility for malaria molecular surveillance worldwide. Additionally, this panel includes 36 amplicons targeting ten candidate genes associated with antimalarial resistance. The second panel, PvCRiSP, consists of four highly-polymorphic amplicons. This high-sensitivity panel is ideal when dozens or hundreds of amplicons do not improve genotype discrimination or enhance detection of minor clones in polyclonal infections. PvCRiSP is best suited for efficiently estimating the number of genetically distinct parasites within a single infection (complexity of infection, or COI); identifying instances of clonal transmission, for example in localized outbreaks; or characterizing recurrent infection episodes in therapeutic efficacy studies. Both panels work on all Illumina sequencing platforms and require a single multiplex PCR reaction prior to the indexing reaction. Both also involve non-proprietary and easily-sourced reagents, facilitating global access and sustainable programmatic use. In this manuscript, we describe the performance of the panels to assess their utility for various use cases.

## Methods

### Ethics statement

All sequenced clinical samples were collected with ethical approval from local institutions. Written informed consent was obtained from participants before clinical samples were collected and in the case of children, formal consent was obtained from parents or guardians. In Colombia, approval was granted by the Comité de Ética para Investigación en Humanos Centro Internacional de Vacunas (CECIV) under the protocol titled “Vigilancia Molecular de la Malaria en Colombia”. In Guyana, approval was obtained from the Ministry of Health Institutional Review Board for protocol 645/2019. In Honduras approval was granted by the UNAH Comité de Ética de Investigación de la Maestría de Enfermedades Infecciosas y Zoonóticas for protocols 02–2023 and 03–2020. In Panama, approval was obtained from Comité de Bioética de la Investigación del Instituto Conmemorativo Gorgas de Estudios de la Salud for protocols 468/CNBI/ICGES/06, 413/CNBI/ICGES/12, and 314/CBI/ICGES/24. In Peru, approval was obtained from UNTRM Comité de Ética de Investigación (CIEI) for protocols CIEI-N° 011, and CIEI-N° 015. In Venezuela, approval was granted by the Comité Independiente de Bioética para la Investigación del Centro Nacional de Bioética Venezuela (CIBI-CENABI) for protocol CIBI-CENABI-03/2022. Sample analysis was approved by the Harvard University Longwood Institutional Review Board (Protocols: IRB19–1779, IRB20–1636, IRB23–0621, IRB23–1567).

### Clinical samples and genomic databases

Multiple genomic data sources and clinical samples were used for the selection, standardization, and validation of PvGTSeq and PvCRiSP amplicons. In the selection process for PvGTSeq amplicons, genomic data from four different sources were used: 1) 1895 genomes from the MalariaGen Pv4 database [21], 2) 89 Brazilian genomes available from the European Nucleotide Archive under project codes PRJEB56411, PRJEB44419, PRJEB36199 [22], 3) 705 genomes obtained from Guyana not present in MalariaGen Pv4 [23], and 4) 532 new genomes obtained from clinical samples from Colombia (n = 200), Peru (n = 90) and Venezuela (n = 342). For the validation of PvGTSeq and PvCRiSP, a total of 821 additional clinical blood spot or DNA samples from Colombia (n = 259), Guyana (n = 460), Honduras (n = 39), Panama (n = 53), and Venezuela (n = 10) were genotyped using one of these methods (S1 Fig).

### Parasite DNA quantification

We measured parasitemia for a subset of clinical samples used in assay optimization, limit of detection measurement (LOD), and minor clone sensitivity analysis. Parasitemia was measured by real-time quantitative polymerase chain reaction (qPCR) using the 18S ribosomal gene [24] and a standard curve from the 3D7 strain of *P. falciparum*. Details about the master mix and cycling conditions are in S1 Text. Due to the lack of a quantified *P. vivax* culture or large volumes of a high-parasitemia sample that could be used as a control for all assays in this study, to quantify *P. vivax* parasitemia on clinical samples we used genomic DNA from the monoclonal *P. falciparum* reference strain, 3D7, as a quantitation standard. The parasite strain was grown using leukocyte depleted red blood cells and we prepared a positive control template representing infected human blood with 10,000 parasites/μL by mixing human genomic DNA (13.76 ng/μL) with 3D7 genomic DNA (0.92 ng/μL) at 2.66:1 ratio (v/v), yielding 10 ng/μL human gDNA and 0.25 ng/μL 3D7 gDNA. The 0.25 ng represents 10,000 *P. falciparum* genomes (based on 23-Mbp genome size and 660 g/mol bp average mass), assuming one haploid genome per infected cell. We created controls representing 10,000, 1000, 100, 10 and 1 parasites/μL by serial 1:10 dilution with 10 ng/μL human gDNA. Because primer pair amplification efficiency may differ between *P. falciparum* and *P. vivax*, we measured the amplification efficiency in the logarithmic PCR phase for each sample as a correction factor relative to the standard curve efficiency.

### Selection of amplicons and primer design

To describe patterns of population structure and connectivity at three geographic scales (global, Latin America, and subnational within Latin America), we selected discriminatory amplicons using Discriminant Analysis of Principal Components (DAPC) [25]. Whole genome sequencing information (WGS) from the MalariaGEN Pv4 dataset [26] and lab-generated WGS were filtered to remove low-quality regions and genotyping error-prone loci. Genomes were divided into 150 bp segments, with segment selection based on: 1) >0.1% contribution to top discriminant components, 2) highest contribution within 200,000 bp windows, and 3) location in functionally annotated genes. Segments in uncharacterized genes were selected only when no other segments met the first criterion within a window. We performed this selection process independently for each of the three geographical scales, generating three subsets of segments. Segments shared across all three subsets were prioritized for primer design.

To confirm that our amplicon panel can detect population structure, we compared the patterns revealed by principal coordinate analysis (PCoA) with those generated using whole-genome data and two previously reported amplicon panels, PvAmpliSeq and rhAmpSeq [14,17,20]. We performed this analysis at two geographic scales: across world regions and among countries in Latin America and the Caribbean. In addition to WGS from MalariaGEN Pv4 [21] and our lab generated genomes, we include 89 Brazilian genomes available from the European Nucleotide Archive [22]. To avoid bias from missing data, we included only samples with WGS information that had ≥ 75% coverage both genome-wide and across the markers for each of the three amplicon panels analyzed. PCoA was performed using the inverse of identity by descent (IBD), with IBD calculated using the R package paneljudge [11,27,28]. For WGS data, IBD was calculated using only biallelic SNPs whose minor alleles are present in at least five samples in the dataset, while for the amplicon panels we used all variant sites (with minor alleles present in at least five samples), excluding those located in homopolymers and dinucleotide short tandem repeats (STRs). We assessed the accuracy of IBD estimates for each amplicon panel by measuring the root mean squared error (RMSE) relative to WGS, and we assessed precision by quantifying the coefficient of variation (CV). We calculated RMSE and CV in 0.05 IBD windows spanning 0–1. We tested differences between amplicon panels in each window using a paired Student’s t-test.

For surveillance of antimalarial drug resistance, we identified 150 bp segments (with 50 bp flanking regions) covering polymorphic sites in 11 candidate resistance genes: *CRT* (PVP01_0109300) [29–35], *MRP1* (PVP01_0203000) [36–39], *DMT2* (PVP01_0312700) [38], *DHFR* (PVP01_0526600) [33,36,38,40–45], *MDR1* (PVP01_1010900) [30,36,45–50], *PI3K* (PVP01_1018600) [49,51], *ABCE1* (PVP01_1103800) [35], *KELCH13* (PVP01_1211100) [33,38,52], *MDR2* (PVP01_1259100) [53,54], *DHPS* (PVP01_1429500) [31,33,44,45,54–58], and *MRP2* (PVP01_1447300) [38,53]. Finally, we contracted the services of GTseek LLC [59] to design multiplexable primer sets following Genotyping in Thousand Sequencing (GT-Seq) protocol specifications [60]. We named this amplicon panel PvGTSeq in honor of its design and its capacity to genotype up to 1,152 samples and 249 amplicons per sample in a single sequencing run using a NovaSeq instrument. We then selected four amplicons from PvGTSeq—**C**G2_related, **RI**PR, VP**S**11, and **P**IGM—to form the PvCRiSP panel. These four amplicons were chosen based on their heterozygosity, ability to detect polyclonal infections, and superior amplification efficiency across the various Latin American countries analyzed with PvGTSeq.

### Whole genome sequencing and variant call

For the 1271 clinical samples from Colombia (n = 459), Guyana (n = 460), and Venezuela (n = 352) we extracted total genomic DNA from dried blood spot samples (20–35 mm^2^ spotted area punched per sample) using KingFisher Ready DNA Ultra 2.0 Prefilled Plates on the Kingfisher Flex instrument (ThermoFisher Scientific). For the 182 DNA samples from DNA Honduras (n = 39), Panama (n = 53), and Peru (n = 90), our collaborators sent DNA they had stored in their sample banks from previous studies. We subsequently applied selective whole genome amplification (sWGA) to DNA extracts [61]. Each 50 μL sample reaction consisting of 5 μL 10x phi29 polymerase buffer (NEB B0269S), 0.125 μL (2.5 μg) recombinant albumin (NEB B9200S), 0.5 μL primer mix (10 oligos combined at 250 μM, see below), 5 μL 10 mM dNTPs (Thermo Scientific), 16.375 μL nuclease-free water, 3 μL (30 units) phi29 DNA polymerase (M0269L), and 20 μL DNA. Reactions were prepared on ice, with components added in the order shown. We used ‘Pvset1’ for *P. vivax* as reported by Cowell et. al. 2017. Amplifications were generated using step down incubation (35 °C for 5 min, 34 °C for 10 min, 33 °C for 15 min, 32 °C for 20 min, 31 °C for 30 min, 30 °C for 16 h, and 65 °C for 15 min), followed by cooling to 4 °C. Note that we applied this sWGA protocol only to obtain whole genome sequencing information. We subsequently modified this protocol to enhance its sensitivity for low parasitemia samples (S1 Text), and that final version which is based on the Genomiphi v2 kit (Cytiva) was used to generate amplicon sequencing data through our PvGTSeq panel. We then applied AMPure XP magnetic beads (Beckman Coulter A63881) at room temperature to exchange post-reaction sample buffer to 10 mM Tris-HCl + 0.1 mM EDTA. Final library construction using the NEBNext Ultra II FS DNA Library Prep Kit (NEB E6177) and 2 × 151 bp sequencing on the Illumina NovaSeq 6000 platform was completed at the Broad Institute. We aligned reads to the *P. vivax* P01 reference genome version 1 [62] assemblies using BWA-MEM v0.7.17-r1188 [63] and called SNPs and INDELs using GATK v3.5-0-g36282e4 [64] ‘HaplotypeCaller’ and ‘GenotypeGVCFs’ according to best practices defined by the Pf3k consortium (http://www.malariagen.net/data_package/pf3k-5/). The mapping and joint variant call process also included samples from the MalariaGEN Pv4 dataset. All downstream genetic analyses focused on variant sites, single nucleotide polymorphisms (SNPs) and insertions and deletions (INDELs), in core regions of the genome. Samples missing genotype calls for >25% variant sites at ≥2% minor allele frequency (after application of the core region filter) were also excluded from further analysis. Following sample exclusions, we removed variant sites located in coding regions prone to alignment errors (coding regions with observed heterozygosity above the 95th percentile and variant site density exceeding the 92.5th percentile of all coding regions in all populations). We also removed variant sites located in homopolymers and di-nucleotide short tandem repeats, as these are prone to PCR errors.

### Panel protocols and optimization

To facilitate standardization of library generation procedures, we started with the conditions described for a previously developed *P. falciparum* protocol [11]. From there, we made modifications to annealing temperature and primer concentrations, the final volume of the multiplex PCR reaction, and the primer sets and enzymes for sWGA. These modifications were applied to improve sequencing yield in terms of the number of on-target reads per sample per amplicon. For this standardization, we selected 60 clinical samples from Colombia (n = 30), Peru (n = 15), and Venezuela (n = 15), each with parasitemia levels above 100 parasites/μL. The final protocol for the library generation and sequencing for PvGTSeq and PvCRiSP can be found in S1 and S2 Texts, respectively. Individual protocol modifications were evaluated in a paired manner, using the previous protocol conditions as a reference. Comparisons were made in terms of the number of reads per sample per amplicon, the total number of reads per sample, and the percentage of amplicons amplified per sample. Additionally, at each step, the rate of non-target product generation for each primer was evaluated, and the primer pairs that generated a higher amount of non-target reads compared to specific reads were removed from the protocol for subsequent optimization rounds (S1 Fig).

### Amplicon data analysis

We used the pipeline previously described for *P. falciparum* with some modifications to generalize its use for *P. vivax* [11]. Briefly, we processed paired-end Illumina sequencing data in the form of FASTQ files using a custom analysis pipeline for which documentation can be found at https://github.com/broadinstitute/malaria-amplicon-pipeline. This pipeline utilizes the Divisive Amplicon Denoising Algorithm (DADA2) [65] to obtain microhaplotypes (i.e., alleles or ‘amplicon sequence variants’). We first aligned microhaplotypes obtained from dada2 against a custom-built database of PvP01 reference sequences for each amplicon locus. We then summarized observed sequence polymorphism into a concise format by converting individual microhaplotypes into ‘pseudo-CIGAR’ strings using a custom python script that can be found at https://github.com/Paulonvnv/MHap-Analysis, The rules used for this conversion are also detailed in S2 Fig. Next, microhaplotypes were discarded if supported by fewer than 10 read-pairs or by less than 1% of total read-pairs within a heterozygous locus. We also filtered out microhaplotypes exhibiting less than 80% identity to the reference sequence. We then masked variants in both the FASTA sequence of the microhaplotype and its pseudo-cigar string representation according to three criteria: 1) SNPs or INDELs located in homopolymer regions (≥5 consecutive identical nucleotides), 2) INDELs positioned at either the beginning or end of the microhaplotype sequence, and 3) variants predominantly (> 66%) present as minor alleles and mainly found in heterozygous sites (> 66%) across the population. All downstream analyses were performed based on the pseudo-cigar strings using custom R scripts that can be found at https://github.com/Paulonvnv/PvGTSeq_PvCRiSP_paper. All functionalities created to handle pseudo-cigar strings are documented at https://github.com/Paulonvnv/MHap-Analysis. This pipeline is also implemented in a Terra workspace (see S1 Text)—a user-friendly cloud-based platform for processing amplicon sequencing data.

### Genotyping performance: Amplification rate and limit of detection (LOD)

Following pseudo-cigar generation and filtration steps, we evaluated the amplification rate and LOD using a set of 160 and 240 samples for PvGTSeq and PvCRiSP, respectively, and from three different endemic countries: Colombia, Guyana, and Venezuela. For PvGTSeq, parasite concentrations ranged from 0.26 to 1309.38 parasites/μL. For PvCRiSP, which contains only four amplicons and was expected to have greater sensitivity, we used lower concentrations. We performed a 1/10 dilution of the samples previously used for PvGTSeq, resulting in final concentrations ranging from 0.03 to 130.94 parasites/μL. This approach ensured sufficient samples with low parasite concentrations that would not amplify any of the four PvCRiSP markers, allowing us to obtain a more precise estimate of its LOD. The amplification rate was analyzed at both the amplicon and sample level, and was defined as the percentage of samples with ≥10 reads per amplicon and as the percentage of amplified amplicons per sample, respectively. The LOD was calculated for each individual amplicon as well as the complete panel of amplicons that pass the optimization step (249 for PvGTSeq and 4 for PvCRiSP). For each amplicon, the LOD was defined as the minimum parasite concentration needed to generate a microhaplotype with ≥10 reads with a 75% probability in the case of PvGTSeq, or 95% in the case of PvCRiSP. LOD at the level of the amplicon was calculated through logistic regression with a quasi-binomial distribution, where parasite concentration was the explanatory variable and success [1] or failure (0) of amplification in the samples was the response variable. The quasi-binomial distribution helped adjust for outliers caused by poor DNA quality or other factors that increase data dispersion not included in the model. Amplicons were classified as highly sensitive if they amplified samples with concentrations <10 parasites/μL, and only this set of amplicons was used for subsequent analysis.

The LOD at panel level was defined as the minimum parasite concentration needed to detect ≥10 reads per microhaplotype at 75% of all amplicons in the case of PvGTSeq and 100% of all amplicons in the case of PvCRiSP. In both cases, LOD was calculated through linear regression between the logarithm of parasite DNA concentration (explanatory variable) and the logarithm of the odds of amplified amplicons per sample (dependent variable).

### Consistency across assays

The consistency of genotyping was determined through reproducibility of the microhaplotype calls between technical replicates of monoclonal infections within the same sequencing run (30 duplicate samples), between sequencing runs (68 duplicate samples), and between genotypes obtained from amplicon sequencing and genotypes extracted from WGS VCF files (39 duplicate samples). Due to differences in filtering parameters for sequencing and alignment errors between GATK and our dada2-based pipeline, no quality filters were applied to the VCF files obtained from joint calling. Instead, VCF files were reconverted into FASTA sequences that were incorporated into our analysis pipeline as if they were reads obtained from amplicon sequencing. Thus, for each technical replicate we calculated the error rate of the sequencing, defined as the percentage of nucleotide differences between replicates with respect to the total number of base pairs across all amplicons.

### Detection of major and minor clones in polyclonal infections

The ability of PvGTSeq and PvCRiSP to detect microhaplotypes from different clones that co-occur within the same infection at different concentrations was measured using mock polyclonal samples generated by combining samples from different geographical areas. Four samples were selected, two from Colombia and two from Guyana, with similar parasite concentrations. The selected clinical samples for generating mock polyclonal infections were the following: SP0112286092, G4GWM400, SP0112286092, and G4GWM400; henceforth we will refer to them as samples S1, S2, S3, and S4 respectively. Samples S1 and S3 were from Colombia and their concentrations were 198.25 and 203.05 parasites/μL, respectively. Samples S2 and S4 were from Guyana and their concentrations were 114.78 and 119.06 parasites/μL, respectively. When analyzing these samples individually through the amplicons from PvGTSeq, samples S1, S2, and S3 were monoclonal, while sample S4 was polyclonal, with a fraction of heterozygous amplicons equal to 0.22 and a maximum number of microhaplotypes per amplicon of 3. The proportion of loci that differed between the haplotypes of samples S1 and S2 was 0.52, while for samples S3 and S4 it was 0.48. The mock sample mixtures generated with samples S1 and S2 were named Mock Sample 1 (MS1), and those generated from samples S3 and S4 were named MS2. Each combination was performed at seven different proportions between the samples (1:10, 1:5, 1:2, 1:1, 2:1, 5:1, and 10:1) and in duplicate, generating 28 mock samples in total. In all mock samples, the parasite concentration of the minority clone was always above the detection limit of the technique, so that the absence of detection should only be explained by competition between alleles during the amplification or sequencing steps.

To evaluate whether the amplicon panels could detect both major and minor clones within polyclonal infections, we calculated the percentage of clone-specific alleles detected (private alleles). This percentage was determined by dividing the number of detected private alleles by the total expected private alleles and multiplying by 100.

### Diversity and utility of the amplicon panels

We subsequently evaluated the ability of both amplicon panels to discriminate whether two infections are identical by state or not in different geographic regions. This analysis only included populations, world regions, or countries with 20 or more monoclonal samples with coverage for at least 75% of the amplicons in the PvGTSeq panel. The genetic sequences of monoclonal samples were obtained from three different sources: 1) WGS from the MalariaGEN Pv4 database, 2) WGS from our laboratory, and 3) amplicon sequencing data from samples sequenced using the PvGTSeq amplicon panel. A monoclonal sample was defined as one that had an *F*_*ws*_ score greater than or equal to 0.975 [66] and a fraction of heterozygous loci less than or equal to 0.05, with loci being defined as variant sites in the case of a VCF file for WGS samples, or as microhaplotypes in the case of samples sequenced by PvGTSeq. To maintain consistency in variant calling between WGS and PvGTSeq sequences, VCF files were converted into FASTA sequences and incorporated into our analysis pipeline as if they were reads obtained from amplicon sequencing.

To determine the diversity of the amplicons in each geographic region, each amplicon was described in terms of the 1) number of polymorphic sites (S), 2) the number of microhaplotypes, and 3) the per-site nucleotide diversity. The nucleotide diversity, or π, was defined as the average number of nucleotide differences per site (each single nucleotide position in the amplicon) between two randomly selected sequences from the population. All these metrics were described independently for the set of amplicons used for geographic attribution at different administrative levels, for amplicons of PvCRiSP and for amplicons covering candidate drug resistance loci. We compared π between amplicon subsets using the Wilcoxon test for unpaired samples, and compared π between geographic regions using the Wilcoxon test for paired samples.

To directly measure the utility of PvGTSeq and PvCRiSP, we measured the capacity of these panels to distinguish between 1) parasites with distinct genomic sequences and 2) parasites that do not belong to the same clonal group. To assess discrimination of genomically distinct parasites, we used WGS data from genetically distinct monoclonal samples (we kept samples with pairwise identity by state or IBS < 0.999 or> ~10 SNPs difference) and samples sequenced only by PvGTSeq. For samples sequenced exclusively by PvGTSeq, we assumed all haplotypes were genomically distinct since they originated from different infected patients. For assessing whether parasites do not belong to the same clonal group, we relied solely on WGS information, defining a clonal group as haplotypes with genetic similarity (IBS) ≥ 0.99 or < 100 SNPs difference in pairwise comparisons. For both scenarios and both amplicon panels, the discriminatory power D (due to its similarity with Simpson’s index) was quantified as the proportion of in silico pairwise sample comparisons from the same population yielding IBS values less than one. IBS was calculated as the proportion of microhaplotypes shared between sample pairs across all amplicons in each panel, or as the proportion of biallelic SNPs shared when comparing WGS information. D was reported for each country, and we selected country as the unit of analysis because it is the smallest geographic unit with sufficient sample size across our dataset, and it is also the context in which most future studies will use these two panels.

### Population structure and geographic attribution

We evaluated the ability of PvGTSeq to detect population structure at three geographic levels (global, intra-continental, and sub-national). PvGTSeq data was generated by applying our amplicon panel to clinical samples or by extracting PvGTSeq coordinates from whole genome sequencing data—either from the MalariaGEN Pv4 database or from our laboratory. At the global and intra-continental levels, population structure was assessed through PCoA, while at the sub-national level, a network analysis was used instead. For both approaches, pairwise genetic similarity between infections was determined using IBS. To avoid introduction of bias due to directional selection [67], amplicons from candidate drug resistance loci, that in most cases also showed low diversity, were excluded from this analysis. We performed Tajima’s D test on each amplicon in each country to confirm that none were under directional selection pressure. To prevent clonal expansions from skewing the results, we selected only one haplotype from each clonal group (IBS > 0.99) in each population. We conducted the analysis only in countries with more than 20 non-clonal haplotypes. We excluded amplicons under strong directional selection pressure (Tajima’s D < -2) from the population structure analysis. However, we reported but did not exclude amplicons under strong balancing selection pressure (Tajima’s D > 2), because this study does not aim to infer historical genealogical relationships among populations—the focus is to observe whether population differentiation exists.

## Results

### Selection of amplicons and protocol optimization

We identified 60,056 variant sites meeting our inclusion criteria: located in coding regions not prone to alignment errors, not in homopolymers or di-nucleotide short tandem repeats. A total of 1,292 genomes had coverage ≥ 75% supported by ≥ 5 reads for these variant sites (S1 Fig). Using DAPC, we identified 263 segments (150 bp each) contributing to population differentiation across three geographic scales (S1 Fig). We also identified 57 non-overlapping segments containing polymorphisms in 11 genes previously associated with antimalarial resistance. From this pool of 320 segments, we successfully designed primers for 271, which underwent successive rounds of protocol optimization. Of these, 249 amplicons amplified efficiently and specifically in the 60 clinical samples used for the standardization step. The final protocol for library generation and sequencing is detailed in the S1 and S2 Texts, and the complete list of 249 amplicons, including primer information and reference genome coordinates, is provided in the S1 Table. The finalized panel consists of 213 amplicons specialized for geographic differentiation (112 for global-scale, 122 for between-country, and 110 for subnational differentiation in Latin America). Note that many of the amplicons nevertheless function at multiple geographic scales.

While we did not evaluate the minimum number of amplicons needed for genetic differentiation between populations, we did assess whether our panel contains enough amplicons to distinguish samples that are genetically different at genome level. In addition, PCoA showed that the 213 amplicons in PvGTSeq reproduces the differentiation patterns observed with WGS and outperforms the other two amplicon panels at both geographic scales analyzed: world regions and countries in Latin America and the Caribbean (S3 and S4 Figs). PvGTSeq successfully distinguished the eight world regions. Within LAC, PvGTSeq distinguished samples from Mesoamerican countries, the Colombian Pacific coast, and the Amazon. However, differentiation between the Amazon and the Guiana Shield was not clear, likely due to the smaller numbers of samples from Venezuela [9] and Guyana [15] compared with other populations (> 20 samples per country). All three amplicon panels showed high accuracy and precision when WGS-based pairwise IBD was close to one (RMSE and CV < 0.1) (S5 Fig). In contrast, when IBD was below 0.5, accuracy and precision declined, with a clear tendency to overestimate IBD relative to WGS (S5A Fig). Among the three methods, PvGTSeq was the most accurate: RMSE was below 0.1 for IBD values above 0.45 and approximately 0.16 when IBD was in the range 0.15–0.45. In this range (0.15 - 0.45), PvGTSeq RMSE was significantly lower than PvAmpliSeq and rhAmpSeq (paired Student’s t-test, p-value < 0.001) (S5B Fig). In contrast, rhAmpSeq was the most precise. rhAmpSeq and PvGTSeq maintained a CV below 0.1 for IBD values above 0.35; however, at lower IBD values, PvGTSeq CV was higher than rhAmpSeq CV (S5C Fig). Because of differences in IBD estimates between the amplicon panel and WGS, we opted to use IBS for subsequent analyses.

The finalized panel additionally targets 36 amplicons within 10 genes associated with antimalarial resistance. The segment identified for the *CRT* gene failed at the primer design stage. For the DHFR gene, we included only the segment spanning amino acids 6–24; other polymorphic regions in this gene are not covered by the final panel. As a result, the previously reported mutations 50I, 57L, 58R, 61M, 117N, and 173L cannot be monitored with this panel [41,42,45,68–71]. For DHPS, the panel includes the 383G mutation and mutations located between amino acids 28–43 and 622–650; however, the 553G mutation fall outside the regions covered by our amplicons [42,56,71]. For *MDR1*, the PvGTSeq panel includes the 976F and 1076L polymorphisms, which have been proposed as markers of chloroquine resistance [46,48,71–76]. The number of amplicons for each candidate antimalarial resistance gene, and the coding regions covered by those amplicons are shown in Table 1. The haplotype frequencies of these 10 genes are shown in S2 Table and in S6 Fig, and we will interpret the temporal and geographic distribution of these haplotypes in a subsequent publication. Finally, we selected four highly polymorphic amplicons from this panel that were heterozygous in most polyclonal samples and showed high amplification efficiency. These four amplicons—**C**G2_related, **RI**PR, VP**S**11, and **P**IGM—form the PvCRiSP panel. All amplicons are named according to their corresponding genes.

**Table 1:** Nucleotides and amino acids in antimalarial resistance candidate genes covered by PvGTSeq.

Gene ID	Gene name	Number of amplicons	Covered nucleotides in the coding sequence	Covered amino acids
PVP01_0203000	*MRP1*	7	755-862, 1930-1983, 2704-2862, 3032-3202, 3673-3853, 4573-4635, 4751-4843	252-288, 644-661, 902-954, 1011-1068, 1225-1285, 1525-1545, 1584-1615
PVP01_0312700	*DMT2*	1	655-663	219-221
PVP01_0526600	*DHFR*	1	17-72	6-24
PVP01_1010900	*MDR1*	5	642-719, 1493-1607, 2698-2750, 2917-3091, 3203-3249	214-240, 498-536, 900-917, 973-1031, 1068-1083
PVP01_1018600	*PI3K*	10	231-354, 551-602, 879-1043, 1270-1325, 2396-2525, 2649-2796, 3350-3407, 3535-3578, 4349-4449, 4695-4745	77-118, 184-201, 293-348, 424-442, 799-842, 883-932, 1117-1136, 1179-1193, 1450-1483, 1565-1582
PVP01_1103800	*ABC-E1*	2	50-106, 1420-1475	17-36, 474-492
PVP01_1211100	*K13*	1	1838-1900	613-634
PVP01_1259100	*MDR2*	4	907-994, 1394-1565, 2525-2578, 3226-3288	303-332, 465-522, 842-860, 1076-1096
PVP01_1429500	*DHPS*	3	84-127, 1115-1172, 1865-1948	28-43, 372-391, 622-650
PVP01_1447300	*MRP2*	2	176-280, 433-547	59-94, 145-183

### Genotyping performance

**Fig 1:**
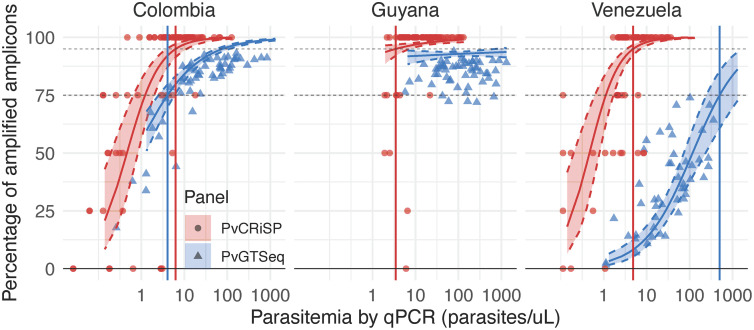
Limit of detection of PvGTSeq and PvCRiSP in Colombia, Guyana and Venezuela. The percentage of amplified amplicons per sample as a function of parasite concentration is illustrated for PvGTSeq (Blue) and PvCRiSP (Red). Each dot represents an individual sample and regression model (solid curve line) was constructed using the logarithm of parasite concentration (independent variable) and the logarithm of the odds of the amplified amplicons per sample (dependent variable) in each country. Vertical lines represent the minimum concentration a sample must have to amplify 75% of the 249 amplicons in PvGTSeq (blue lines) or the four amplicons of PvCRiSP (red lines). The shaded area between the dashed curve lines represents the 95% confidence interval.

The amplification rate of the amplicons and the limit of detection varied among the three populations analyzed with PvGTSeq due to sample quality, but PvCRiSP yielded consistently high performance across populations. Regarding PvGTSeq and using the MiSeq Illumina instrument, in Colombia, 190 out of the 249 amplicons amplified in more than 75% of the samples (S7A and S7B Fig and S3 Table), with a median read depth of 95.5 (interquantile range or IQR = 409) across the 249 amplicons. The median LOD of these amplicons analyzed individually was 4.64 (IQR = 14.6) (S7C and S7D Fig), and the minimum parasite concentration (LOD) to amplify 75% of the total 249 amplicons was 4.05 parasites/μL (95% CI from 2.5 to 5.9) (Fig 1). When considering only the amplicons that worked in more than 75% of the samples in Colombia, the LOD to amplify 95% of these 190 amplicons was 3.24 parasites/μL (95% CI from 1.18 to 6.15) (S4 Table). A similar result was obtained with samples from Guyana, where 201 amplicons amplified in more than 75% of the processed samples; however, parasite concentrations were above 5.73 parasites/μL, and LOD could not be determined either at the individual amplicon level or for the complete panel. Furthermore, there were 201 amplicons that had amplification rates above 75% in Colombia or Guyana. In contrast, Venezuelan samples showed poor amplification success, where only 21 amplicons were amplified in more than 75% of the samples.

We next investigated reproducibility, ability to detect minor alleles in polyclonal infections and population structure signals using the group of 201 amplicons that showed over 75% amplification success in Colombia or Guyana to avoid bias from missing data.

The PvCRiSP mini multiplex exhibited similar amplification success and LOD among the three populations using the iSeq Illumina instrument. The four amplicons amplified on average more than 86% of the samples and had a median read depth of 131 (IQR = 446). The amplicon *CG2*_*related*_ showed the lowest amplification rate (77%) and the lowest read depth (median = 45.5, IQR = 127.25) across the three populations (S5 Table). The minimum concentration needed to amplify the four amplicons was 6.33 parasites/μL (95% CI from 4.92 to 8.23) and no significant differences were observed between the three countries when comparing their confidence intervals (S4 Table).

### Consistency across sample replicates

The results showed high reproducibility between technical replicates of monoclonal infections. Within the same sequencing run, of the 30 technical replicates analyzed, only in three cases were there differences between any of the amplicons between replicates. In these three cases, the difference was only one nucleotide between replicates. In two cases, the discrepancies were found in a marker against the gene *RAMA* (PVP01_0107500). In both of these two cases one of the replicates had a haplotype similar to the reference strain PvP01 while the other replicate contained only one non-synonymous polymorphism with respect to the reference. These two polymorphisms were present in 58 and 43 samples respectively in our group of samples used for the estimation of LOD, an indication of cross-contamination. The third discrepancy was a singleton polymorphism in our full data set of sequenced samples that could be caused by a PCR or sequencing error. The error rate including only the genotyping error was 3.85e-4% (phred Q34; permutation test, 95% CI 7.93e-5% to 1.12e-3%).

When comparing replicates between different sequencing runs, we observed that 16 out of the 68 replicates differ in at least one amplicon. Most discrepant cases consisted of a single nucleotide polymorphism (11 cases). Other discrepant cases included the occurrence of two single nucleotide variants (SNVs) in a single amplicon (1 case), one INDEL plus one SNV in a single amplicon (2 cases), one INDEL and one SNV in two different amplicons (1 case), and one INDEL plus three SNVs in a single amplicon (1 case). Moreover, in five cases the discrepancy was in an amplicon that was heterozygous in one of the two replicates. Based on these observed discrepancies between replicates that were not likely caused by contamination the error rate was 1.42e-3% (phred Q28.5; 95% CI from 8.53e-4% to 2.21e-3%).

PvGTSeq panel replicates showed greater differences when compared with WGS replicates (S8 Fig). Thirty-two out of 39 replicates showed differences in one or more amplicons and 30 out of them were due to an INDEL. Most of these INDELs occur in amplicons for seven genes: PVP01_0109700, PVP01_0304700 (LISP2), PVP01_0530700, PVP01_1018600 (PI3K), PVP01_1240400 (ApiAP2), PVP01_1421800, PVP01_1465800. Our amplicons are approximately 150 base pairs, and our sequencing uses 300 total cycles. This allows complete coverage and overlap of the amplicon by both reads generated. Therefore, these discrepancies were explained by inconsistent representation of INDELs in the variant calling process of WGS data. Excluding amplicons against these seven genes, the error rate was 2.87e-3% (phred Q25.4; 95% CI from 1.75e-3% to 4.44e-3%).

### Detection of major and minor clones in polyclonal infections

Next, we tested the utility of the 201 highly sensitive amplicons from PvGTSeq and the four highly sensitive amplicons of PvCRiSP for detecting minor alleles in mock polyclonal infections. The selected clinical samples were named S1, S2, S3, and S4. We mixed S1 and S2 to generate mock samples at seven different proportions between the samples (1:10, 1:5, 1:2, 1:1, 2:1, 5:1, and 10:1) in duplicates, and we labeled those mixtures as Mock Sample 1 (MS1). The mixtures generated from samples S3 and S4 were labeled as MS2. Additionally, the combinations were performed at a total parasitemia of 100.33 parasites/μL for MS1 and 58.46 parasites/μL for MS2 such that the concentration of the minority clone was always above 5.85 parasites/μL, therefore the absence of amplification of the clone is expected to reflect competition between alleles in PCR and/or sequencing, not the clone occurring below panel detection limits.

**Fig 2:**
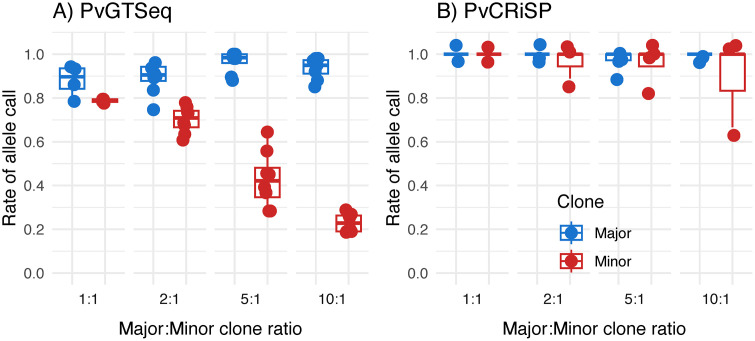
Detectability of alleles from major (blue) and minor (red) clones in mock samples using PvGTSeq (A) and PvCRiSP (B) panels. The X-axis represents the ratio of the concentration of the major clone (numerator) with respect to the minor clone (denominator) in the mock sample. The Y-axis represents the proportion of private alleles of the clone of interest detected (numerator) with respect to the total number of private alleles expected (denominator). Dots represent each clone of interest in the 28 mock samples.

The PvGTSeq and PvCRiSP panels identified all mock samples as polyclonal, including those samples in which the major clone was in a 10:1 ratio compared to the minor clone (Fig 2). In PvGTSeq, the percentage of private alleles detected was > 76% whenever the major clone was in a proportion of 1:1, 2:1, 5:1, or 10:1 relative to the other clone present in the sample. However, for the minor clone, the detection rate of its private alleles decreased linearly with respect to its proportion in the sample (Fig 2A). Thus, only when both clones were in similar proportions in the sample was the observed fraction of heterozygous loci close to the expectation (75%) (S9A Fig), and as the ratio between the major clone to the minor clone increased, the ratio between the observed and expected fraction of heterozygous loci decreased, reaching only 30% when the major:minor clone was in a 10:1 ratio. Additionally, the read depth of the major clone was consistently higher than the read depth of the minor clone across all analyzed amplicons and all technical and biological replicates (S10 Fig). Thus, a positive correlation was observed between the median ratio of read depth of private alleles between major and minor clones with respect to the ratio of DNA input concentrations per clone (S9C Fig). In the case of PvCRiSP, the detection rate of private alleles was always above 67%, even when the major:minor clone ratio was 10:1 (Fig 2B). Therefore, the ratio between observed and expected fraction of heterozygous loci was always one regardless of the proportion of clones within the mock sample (S11B Fig). This result is similar to that obtained with the 4CAST panel designed for *P. falciparum*, where the sensitivity to detect alleles from the minor clone was 94% even with ratios of 10:1 [11].

### Diversity and utility of the amplicon panels

**Fig 3:**
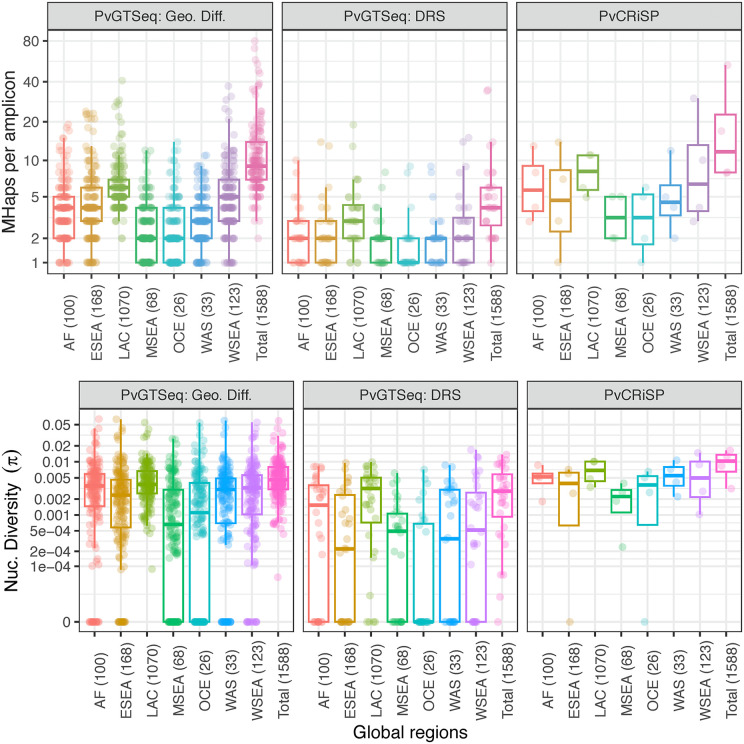
Number of microhaplotypes (MHaps) and nucleotide diversity (π) per amplicon use groups and across seven global regions: Africa (AF), Eastern Southeast Asia (ESEA), Latin America and Caribbean (LAC), Maritime Southeast Asia (MSEA), Oceania (OCE), Western Asia (WAS), and Western Southeast Asia (WSEA). Numbers within parenthesis indicate the number of monoclonal clinical samples. Each dot represents an amplicon. Use case groups were defined as 169 amplicons for geographic differentiation, 32 amplicons for drug resistance surveillance (DRS), and the four amplicons comprising PvCRiSP.

Genetic diversity varied across amplicon subsets and geographic areas at different spatial scales. Globally, from the 201 highly sensitive amplicons in PvGTSeq, the median number of polymorphic sites (S, which includes SNVs and INDELs) and microhaplotypes per amplicon was 7 (IQR = 6) and 9 (IQR = 7), respectively. The nucleotide diversity (π) was 4.38e-03 (IQR = 5.13e-03) (Fig 3). Among the four PvGTSeq amplicon subsets (differentiation at three geographical scales and genes associated with antimalarial resistance), those selected for their discrimination power between world regions and within Latin America and Caribbean countries showed the highest diversity (Wilcoxon Rank-Sum p-value < 0.005 with respect to the other subsets) with no significant differences (p-value > 0.005) observed between these two subsets (S11 Fig). The sub-set of amplicons for differentiation between world regions showed a median 9 microhaplotypes (IQR = 7), and a median nucleotide diversity of 4.95e-03 (IQR = 5.04e-03) while in the sub-set of amplicons for differentiation within Latin America and Caribbean countries, the median number of microhaplotypes and nucleotide diversity were 11 (IQR = 12.5) and 5.61e-03 (IQR = 5.58e-03), respectively. Conversely, amplicons targeting antimalarial resistance genes showed significantly lower diversity compared to the other subsets (p-value < 0.05, S = 3, number of microhaplotypes = 4, π = 2.8e-03) (Fig 3), except for two amplicons, pvpi3k_7 and pvpi3k_13, targeting the *PI3K* gene (PVP01_1018600) which exhibited more than 30 microhaplotypes due to short tandem repeats in their sequences. While only the amplicon pvpi3k_1 was monomorphic across all global monoclonal samples, it showed polymorphism in three polyclonal samples from Cambodia, Thailand and Myanmar. Regarding PvCRiSP, it showed more than six polymorphic sites (median = 8.5, IQR = 2.75) and eight microhaplotypes per amplicon (median = 13, IQR = 17.25), with global nucleotide diversity above 3.15e-03 (median = 1.06e-02, IQR = 6.55e-03).

Across the seven global regions analyzed (East Asia was excluded due to its low number of samples), the median nucleotide diversity of the 169 amplicons for geographic differentiation of the PvGTSeq panel exceeded 3.01e-03 in Africa (AF), Eastern Southeast Asia (ESEA), Latin America and Caribbean (LAC), Western Southeast Asia (WSEA) and Western Asia (WAS). Significant inter-regional differences (Wilcoxon Signed-Rank p-value < 0.005) were observed except between AF and WSEA, ESEA and WAS, and WSEA and WAS (Fig 3). Maritime Southeast Asia (MSEA) and Oceania (OCE) showed lower nucleotide diversity (1.75e-03 and 1.7e-03, respectively, p-value < 0.005), with up to 73 (36%) monomorphic amplicons in these regions. This pattern persisted across various PvGTSeq panel subsets (use groups) as well as in the PvCRiSP panel, and the amplicon against the gene *PIGM* in PvCRiSP was monomorphic in OCE. This amplicon was also monomorphic in ESEA. At the country level, Brazil showed the highest nucleotide diversity (π = 5.01e-03, p-value < 0.005) while Malaysia showed the lowest (π = 5.72e-04, p-value < 0.005; S12 Fig). Indonesia and Papua New Guinea were excluded from the country level analysis due to insufficient monoclonal samples, but they were kept at the world region level analysis.

**Fig 4:**
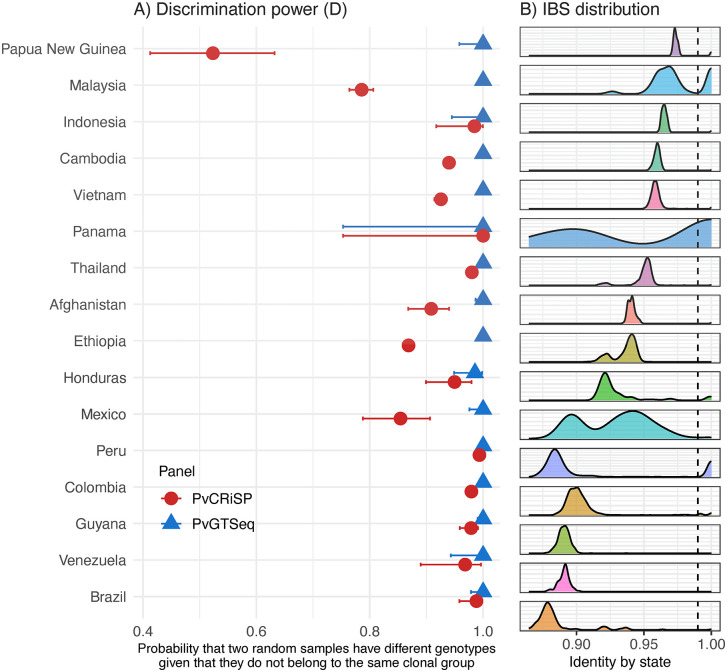
Panel discrimination power. Panel A on the left shows the probability that two random samples have a different genotype (differ in at least one amplicon) given that they do not belong to the same clonal group (x-axis) across global *P. vivax* populations (y-axis) using PvGTSeq (blue) and PvCRiSP (red). Panel B) on the right shows the distribution of identity by state within each population measured from whole genome sequencing data and the vertical dashed line indicates the threshold used to define clonal groups with whole genome information (IBS ≥ 0.99).

**Fig 5:**
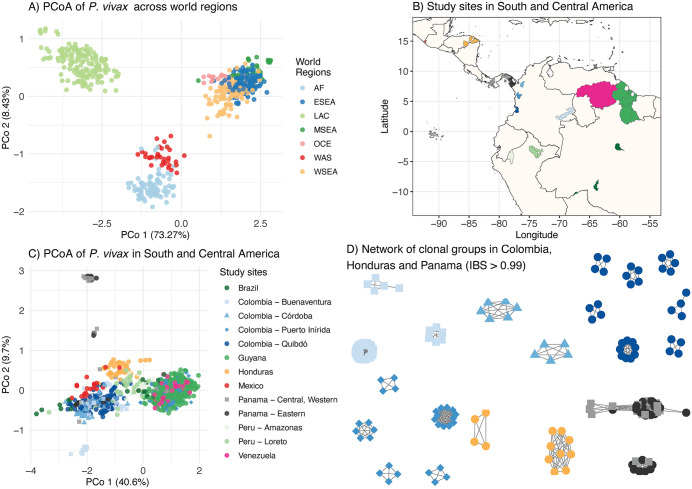
Population subdivision with PvGTSeq. (A)‌‌ Principal coordinate analysis (PCoA) of *P. vivax* samples from seven global regions: Africa (AF), Eastern Southeast Asia (ESEA), Latin America and Caribbean (LAC), Maritime South-East Asia (MSEA), Oceania (OCE), Western Asia (WAS), and Western South-East Asia (WSEA). LAC samples were sequenced either by WGS or the PvGTSeq amplicon panel or were obtained from the MalariaGEN Pv4 database. Genetic information for other regions was extracted from the MalariaGEN Pv4 database. Each point represents a monoclonal sample, with colors indicating geographical origin. The x- and y-axes represent principal coordinates 1 and 2, respectively. B) *P. vivax* sampling areas across South and Central America for genetic characterization using PvGTSeq. Colored regions show the administrative areas where samples were collected—first subnational level for Guyana, Mexico and Venezuela, and second subnational level for Brazil, Colombia, Honduras, and Peru. The map was produced using ggplot2 in R [77,78], and shape files were obtained from the Database of Global Administrative Areas (https://gadm.org/), under CC BY 4.0 using R package GADMTools [78,79]. C) PCoA of *P. vivax* populations in South and Central America. Points represent monoclonal samples, with colors and shapes indicating country of origin, except for Colombia, where colors represent municipality (second subnational level). The x- and y-axes represent principal coordinates 1 and 2, respectively. D) Network graph showing genetic relationships (IBS) among samples within clonal groups (IBS > 0.99) in Colombia, Honduras, and Panama. Each node represents a monoclonal sample, with edges indicating genetic relationships exceeding 0.99 IBS. Panels B, C and D share the same color scheme.

To directly measure the utility of PvGTSeq and PvCRiSP in each country, we defined the discriminatory power of the panels as the ability of: 1) differentiating parasites given that they have different genomic sequences (S13 Fig), and 2) differentiating parasites given that they do not belong to the same clonal group (Fig 4). Regarding the ability of the panels to differentiate parasites with different genomic sequences, for PvGTSeq, it was found that in 15 of the 16 analyzed countries, D was ≥ 0.95, but lower in Panama (0.767, 95% CI from 0.748 to 0.785) (S13 Fig), a country where transmission is dominated by two highly clonal groups (Fig 5D). For PvCRiSP, D was ≥ 0.95 in seven countries, between 0.9 and 0.95 in four countries, and below 0.9 in five countries (Ethiopia, Mexico, Malaysia, Panama and Papua New Guinea). In both panels, D was influenced by the distribution of IBS of the pairwise comparisons in the population (Pearson correlation p-value < 0.001, adj-R^2^ = 0.71).

Because D is associated with the IBS distribution in the geographic unit of interest (diversity and degree of clonality of the population), we redefined D as the probability that two random samples have different genotypes given that these two infections do not belong to the same clonal group. Under this definition, PvGTSeq demonstrated high discriminatory power in all analyzed countries. D was 1.0 for all countries except Honduras, where it was 0.986. For PvCRiSP, D was above 0.8 for all countries, but remained limited in Malaysia (0.786) and Papua New Guinea (0.523). In these two countries, the amplicon targeting *PIGM* was monomorphic and the amplicon targeting *VPS11* has only 2 alleles, reducing the ability of PvCRiSP to discriminate between genomically similar samples or between samples that come from endemic areas with low population diversity.

### Population structure and geographic attribution

None of the amplicons used for the population structure analysis showed Tajima’s D values below -2, and only the amplicon targeting gene PVP01_0834800 showed a value above 2 in Guyana (S14 Fig). Across all countries, amplicons targeting *MRP1* (PVP01_0203000), *MDR1* (PVP01_1010900), *PI3K* (PVP01_1018600), *ABC-E1* (PVP01_1103800), *K13* (PVP01_1211100), and *DHPS* (PVP01_1429500) had Tajima’s D values below -1. Genetic differentiation using a PCoA and a network method revealed that genetic differences align with administrative boundaries at three geographical levels: global, intra-continental, and subnational. The global PCoA divided samples into three major groups: the first composed of LAC, the second including AF and WAS, and the third comprising ESEA, MSEA, OCE and WSEA. Each of these groups showed internal differentiation patterns (Figs 5A and S15). In Latin America and the Caribbean, PCoA analysis revealed that the first principal coordinate, explaining 43% of genetic differentiation, separates parasites from the Guiana shield region (Eastern Colombia, Guyana, and Venezuela) from those in the Colombian Pacific and Central America (Fig 5C). The second coordinate distinguished parasites from Colombia from those in Honduras and Panama. Network analysis of samples from Colombia, Honduras, and Panama further reveals distinct clonal groups specific to each sub-national region (Figs 5D and S16). Finally, the sequencing method used (amplicon or WGS) did not cause large batch effects (S17 Fig).

## Discussion

We describe two new amplicon-based targeted sequencing panels for *P. vivax*, PvGTSeq and PvCRiSP. PvGTSeq is a multipurpose panel that requires sWGA pre-amplification and contains 201/249 highly sensitive amplicons for monitoring variants of interest in nine genes associated with antimalarial resistance (32/36 amplicons) and identifying population structure (169/213 amplicons), suitable for use in any global region but particularly optimized for Latin America. All amplicons are multiplexed in a single PCR reaction using non-proprietary reagents. With sWGA, PvGTSeq offers high sensitivity and amplifies ≥ 75% of amplicons in samples with parasitemias as low as four parasites/μL. The PvCRiSP panel consists of only four highly polymorphic amplicons and works with parasitemias ≥ 5 parasites/μL without sWGA, enabling characterization of samples with compromised DNA integrity. PvCRiSP is tailored towards estimating COI and distinguishing between infections caused by distinct clonal groups, information useful for characterizing outbreaks. We validated both panels directly with samples from five Latin American countries (Colombia, Guyana, Honduras, Panama, and Venezuela) and *in silico* across 16 countries worldwide. This information serves as a reference for potential users to evaluate the utility of these panels in their endemic areas of interest.

The cost per sample of PvGTSeq library preparation and sequencing is $23.64 with 186 samples per MiSeq v2 kit 300 cycles (median of >200 reads/amplicon/sample), plus $15.99 per sample for sWGA using the Genomiphi v2 kit (Cytiva). In contrast, PvCRiSP costs $19.04 per sample due to its simplified design (S6 Table). The number of samples that can be multiplexed in these two panels depends on the Illumina instrument, the i5 and i7 indexes, and the desired average read depth per amplicon (e.g., > 200 reads). For the PvGTSeq, in the iSeq we can multiplex up to 96 samples including controls with a read depth above 200 reads per amplicon per sample. For the MiSeq, we can multiplex up to 192 samples and controls. When using a HiSeq or NovaSeq instrument, the number of samples depends on the i5 and i7 indexes. We use up to 1,152 unique combinations of these indexes—the maximum number of samples (including controls) we can multiplex in a single sequencing run.

These panels contribute to the expanding repertoire of targeted next-generation sequencing approaches for *P. vivax* characterization, all of which have strengths and weaknesses for various use cases. The most common applications of targeted sequencing include analyzing population structure, determining geographic origin, tracking resistance variants, measuring COI, determining pairwise relatedness, and distinguishing between new infections, relapses, and recrudescences [13,14,16–19,80,81]. Spatial resolution depends on the genomic dataset used for target selection and the criteria applied to maximize resolution per area of interest. While MalariaGEN Pv4 data has enabled new panel development, some regions remain underrepresented—particularly Latin America—causing variable panel performance across regions. PvAmpliSeq, the first validated amplicon sequencing panel, was designed to identify patterns of population structure between global regions and within Vietnam [14] but showed poor discrimination in South America, leading to a second version with 41 additional SNPs for Peruvian samples. A recent MIP-based panel targeting ~1200 SNPs tested with Peruvian samples offers deep profiling of samples but struggles with parasitemias below 100 parasites/μL [18]. Others have grouped markers from multiple panels to characterize autochthonous *P. vivax* cases that occurred in the United States in 2023 [82]. While previous panels were designed to characterize SNPs, two additional panels were developed to amplify microhaplotypes (multiple polymorphic sites within a single amplicon), though these had constraints with low parasitemia samples [20,81]. In creating our new PvGTSeq and PvCRiSP panels, we incorporated extensive WGS data from MalariaGEN Pv4 (genomes from Brazil, Colombia, Mexico, and Peru) and we additionally leveraged in-house WGS representing Colombia, Guyana, Peru, and Venezuela. Then we validated both panels with performed samples from Colombia, Guyana, Honduras, Panama and Venezuela. Additionally, we coupled PvGTSeq library preparation with sWGA. As a result, these two panels are the most sensitive and extensively evaluated in the Americas.

Distinguishing between new infections, relapses, and recrudescences requires high sensitivity to detect alleles from minority clones in polyclonal infections. In this context, panels with large numbers of multiplexed amplicons present a significant disadvantage [19,80]. Our results show that while PvGTSeq can correctly identify whether an infection is monoclonal or polyclonal, alleles from the minority clone may remain undetected up to 70% of the time when the proportion between clones is 10:1. Although reducing the number of samples per sequencing run could increase read depth and minimize allele loss from minority clones, this approach would raise costs, making this technology prohibitively expensive. This challenge is effectively addressed by using panels with fewer amplicons, such as PvCRiSP, which offers high sensitivity, efficiently detects all alleles present in polyclonal samples without requiring sWGA, and offers low procedural complexity.

As amplicon sequencing becomes more widespread for generating genetic data for malaria parasites and other pathogens, there is a need for versatile analysis tools that work with any amplicon panel. Here we used a dada2-based analysis pipeline for denoising PCR errors and achieving sensitive minor allele detection in polyclonal infections. We validated our error-filtering approach using technical replicates at three levels: within runs, between runs, and against WGS. This multi-level validation achieved lower error rates (3.85e-4% within sequencing runs and 2.86e-3% when comparing amplicon sequencing to WGS) than comparable studies (from 0.008% to 0.02%) [10] and demonstrated that INDELs do not reduce reproducibility in amplicon sequencing. This indicates that a genotyping error will occur approximately once every 142,000 base pairs or one error every five genotyped samples with the full set of 249 amplicons—a rate comparable to non-proofreading polymerases. Because 36 of the amplicons target genes associated with antimalarial resistance, approximately one in every 50 analyzed samples will have a random error in one of these amplicons. These errors can be filtered out by establishing allele frequency thresholds at the population level (e.g., discarding singletons) or by using technical replicates in therapeutic efficacy studies with few samples. This panel-agnostic pipeline is available on GitHub and embedded in a Terra workspace—a cloud-based platform designed for users with limited bioinformatics expertise (S1 Text). A comprehensive description of this analysis pipeline and all its functionalities will be presented in a subsequent publication.

Despite their significant advantages, both PvGTSeq and PvCRiSP have limitations. These include ascertainment bias inherent in the design of any targeted sequencing panel, limited overlap with existing panels, PvGTSeq allelic dropout in polyclonal samples with skewed strain ratios, and lack of representation of some antimalarial resistance markers. However, despite these limitations, PCoA and network analysis confirm that PvGTSeq can identify population structure on a global scale as well as detect clonal expansions at subnational levels in Colombia and Panama, where persistent clonal groups have been previously documented using WGS [83,84].

While in this work we report the diversity, neutrality and the discriminatory power of our amplicons in up to 16 countries, it is important to note that these analyses were performed with genetic information from MalariaGEN Pv4 or clinical samples provided by our collaborators. Because sampling intensity, strategy, and sequencing criteria may vary among these datasets, we limit our interpretations to the technical applicability of these tools—specifically, whether the amplicon panel is diverse enough to characterize a specific population—and not to comparisons of parasite diversity among populations. We recommend that the use of these or other amplicon panels must be evaluated on a case-by-case basis in future studies. We do not recommend using this panel, or any targeted genotyping panel, for selection screening or demographic inference due to ascertainment bias. Most amplicons were designed to maximize variant sites using data that may not reflect diversity where the panel is implemented. Whole genome sequencing remains the best method for population genetic inference. This highlights the importance of generating and maintaining representative genomic databases over time. Such databases will help to evaluate existing panels based on the context they are intended to be used and guide panel redesign when necessary. To achieve this, it is essential to define representative sampling for different endemic areas and establish criteria for how frequently this information should be generated.

PvGTSeq does not include amplicons targeting the *CRT* gene. This gene is considered a candidate resistance marker due to the role of its ortholog in *Plasmodium falciparum* [71]. In *P. vivax*, this gene has few polymorphisms at very low frequency, and none have been associated with chloroquine resistance [32,46]. However, overexpression of this gene may mediate chloroquine resistance through an increase in the number of TGAAGH motif repeats in the 5’ upstream region or through a deletion within intron nine [85]. To date, no existing panels include amplicons targeting these noncoding motifs, and the original PvGTSeq design excluded these regions as well. PvGTSeq also does not cover all reported polymorphisms in *DHFR* and *DHPS* genes, which are associated with resistance to pyrimethamine and sulfadoxine, respectively. Although these two drugs are not part of first-line treatment for *P. vivax* due to the widespread of mutations conferring resistance in *P. vivax* populations, monitoring them remains important because, in some Sub-Saharan African Counties where *P. falciparum* and *P. vivax* are co-endemic, sulfadoxine-pyrimethamine is still used for *P. falciparum* intermittent preventive treatment in pregnant women and seasonal chemoprevention for children [86]. Primers were designed for all polymorphic regions in these genes; however, some exhibited low sensitivity and specificity in multiplex PCR. Future redesigns may address this by including them in a separate PCR1 reaction (not tested in this study) and subsequently combining the PCR1 products for PCR2 [14,17,18]. This approach highlights the flexibility of PvGTSeq, which utilizes open-source reagents and primers that users can obtain independently.

The results presented here demonstrate that PvGTSeq and PvCRiSP are two highly sensitive panels that allow genetic characterization of infections above five parasites/μL. Through analysis of samples from five endemic countries in Latin America and the Caribbean, we demonstrate that PvGTSeq enables monitoring of most candidate antimalarial resistance variants and identification of population structure at different geographic scales. Meanwhile, PvCRiSP provides reliable estimation of COI and identifies cases of clonal transmission even in samples with compromised DNA integrity. This information will serve as a reference for describing demographic changes in the *P. vivax* population, particularly in Latin America and the Caribbean—a region that in recent years has faced significant challenges for malaria control and elimination.

## Supporting information

S1 Fig Flowchart of the selection, optimization and validation process for PvGTSeq and PvCRiSP. Each stage details the number of amplicons (segments or primer pairs) selected, the samples or genomic data used for the analysis, and the evaluation criteria applied.
(TIFF)

S2 Fig Examples of Pseudo-cigar representation of polymorphisms observed from multiple alignment of amplicon microhaplotypes with their reference sequence. In our analysis pipeline, after dada2 denoising of sequencing errors, all microhaplotypes are aligned to the reference sequence using MUSCLE (Multiple Sequence Comparison by Log-Expectation). Polymorphisms are summarized in Pseudo-cigar format following these rules: 1) All variants are annotated in ascending order. 2) For SNVs, we annotated the reference position followed by the substitute nucleotide. 3) For deletions, we annotated the starting position, “D=”, then all deleted nucleotides (e.g., 23D = TG). 4) For insertions, we annotated the position before insertion, “I=”, then the nucleotide at that position followed by inserted nucleotides. 5) If the nucleotide before an insertion is an SNV, we include this SNV within the insertion notation to avoid position duplication (see MHAP05). 6) When a deletion is followed by an insertion, the insertion is annotated first to maintain ascending order (see MHAP03 and MHAP04). 7) For insertions before position 1, we use position 0 and only annotate inserted nucleotides (see MHAP05-MHAP07). 8) If both reference and microhaplotype have deletions, but the microhaplotype deletion extends beyond the reference deletion, we consider it a single deletion (see MHAP06 and MHAP07). 9) If both, the reference and the microhaplotype, have deletions, but the reference deletion extends beyond the microhaplotype deletion, we consider it a single insertion (see MHAP05). 10) Microhaplotypes that are identical to the reference sequence are annotated using a period symbol “.” (see MHAP01).
(TIFF)

S3 Fig Principal coordinate analysis of world regions comparing whole genome sequencing (A), PvGTSeq (B), PvAmpliSeq (C) and rhAmpSeq (D). The analysis was done using genomic information from MalariaGEN Pv4, Brazilian genomes available from the European Nucleotide Archive (PRJEB56411, PRJEB44419, PRJEB36199) and 112 genomes from Colombia, Guyana, Honduras, Panama, Peru and Venezuela generated in our group. The analysis at world regions scale includes: Africa (AF), East Asia (EAS), Eastern Southeast Asia (ESEA), Latin America and Caribbean (LAC), Maritime South-East Asia (MSEA), Oceania (OCE), Western Asia (WAS), and Western South-East Asia (WSEA).
(TIFF)

S4 Fig Principal coordinate analysis of Latin America and Caribbean *P. vivax* populations comparing whole genome sequencing (A), PvGTSeq (B), PvAmpliSeq (C) and rhAmpSeq (D). The analysis was done using genomic information from MalariaGEN Pv4, Brazilian genomes available from the European Nucleotide Archive (PRJEB56411, PRJEB44419, PRJEB36199), and 112 genomes from Colombia, Guyana, Honduras, Panama, Peru and Venezuela.
(TIFF)

S5 Fig Deviation of IBD estimates from the amplicon panels relative to WGS. A) Scatter plot of IBD estimates for pairs of samples using WGS (x-axis) and the amplicon panels (y-axis). Each point represents a pairwise comparison, and the diagonal line indicates the expected value when IBD estimates from WGS and the panels are the same. B) Root mean squared error of IBD estimates from the amplicon panels relative to WGS across different IBD ranges. Confidence intervals were constructed assuming a chi-squared distribution. C) Coefficient of variation of IBD estimates from the amplicon panels relative to WGS across different IBD ranges. Confidence intervals were constructed assuming a T distribution.
(TIFF)

S6 Fig Bar plot showing the prevalence of haplotypes of genes that carry mutations associated with antimalarial resistance. y-axis shows the frequency in each population, x-axis shows the country in which the sample was collected, horizontal sections correspond to the analyzed gene, and vertical sections represent each world region. (TIFF)

S7 Fig A) Scatter plot of the median read depth and the percentage of amplified samples of each amplicon on each of three populations: Colombia (Green), Guyana (Red) and Venezuela (Blue). Dots represent each of the 249 amplicons in PvGTSeq while the letters represent the 4 amplicons in PvCRiSP (CG2_releated, RIPR, VSP11, and PIGM). B) Distribution of amplification rate of the amplicons in PvGTSeq and PvCRiSP in the three countries, as indicated by color. Numbers in panel B indicate the number of amplicons in each population that are in the 1st, 2nd, 3rd, and 4th quantile. C) Distribution of limit of detection (LOD) of each individual amplicon in PvGTSeq and PvCRiSP. D) Scatter plot of amplification rate (x-axis) and LOD (y-axis) of each individual amplicon (dots or letters) in three populations.
(TIFF)

S8 Fig Contribution of each amplicon and the type of polymorphism to the discrepancies within and between PvGTSeq sequencing runs, and between PvGTSeq with respect to WGS. X-axis shows the number of times an amplicon (y-axis) showed a discrepancy between technical replicates in any of the 3 experiments (Vertical panels): Within and between sequencing runs of PvGTSeq, and between PvGTSeq and WGS. Colors indicate if the discrepancy in the amplicon was due to a single nucleotide variant (SNV, in gold), an insertion or deletion (INDEL, in red) or any of both (in blue).
(TIFF)

S9 Fig The top panels show the fraction of heterozygous loci detected relative to the ratio of major and minor clones in mock samples using PvGTSeq (A) and PvCRiSP (B). The bottom panels illustrate the correlation between the read depth ratio of major and minor clones for PvGTSeq (C) and PvCRiSP (D) compared to their DNA concentration ratios. MS1 and MS2 represent the two biological replicates, and shapes of the dots (circles and triangles) represent the 2 technical replicates.
(TIFF)

S10 Fig Read depth of major and minor clones within mock samples. Each dot represents a private microhaplotype from major (blue) and minor (red) clones using the 201 amplicons from PvGTSeq. Horizontal panels represent each of the two combinations of mock samples (MS1 and MS2) and their technical replicates (R1 and R2), while the vertical panels represent the different ratios at which mock samples were generated.
(TIFF)

S11 Fig Number of polymorphic sites, microhaplotypes (MHaps)alleles and nucleotide diversity (π) per amplicon use groups and across seven global regions: Africa (AF), Eastern South-East Asia (ESEA), Latin America and Caribbean (LAC), Maritime South-East Asia (MSEA), Oceania (OCE), Western Asia (WAS), and Western South-East Asia (WSEA). Numbers within parenthesis indicate the number of monoclonal clinical samples. Each dot represents an amplicon. Each dot represents an amplicon. Use case groups were defined as 81 amplicons for geographic differentiation between world regions (WR), 49 amplicons for geographic differentiation between countries in LAC (LACbc), 35 amplicons for geographic differentiation within countries in LAC (LACwc), 32 amplicons for drug resistance surveillance (DRS), and the four amplicons comprising PvCRiSP. Because there are amplicons for geographic differentiation that belong to multiple groups, to avoid duplication we assigned the amplicon to the highest geographical scale.
(TIFF)

S12 Fig Number of polymorphic sites, microhaplotypes (MHaps) and nucleotide diversity (π) per amplicon use-case groups and across 13 countries: Ethiopia (ETH), Cambodia (KHM), Vietnam (VNM), Brazil (BRA), Colombia (COL), Guyana (GUY), Honduras (HND), Panama (PAN), Peru (PER), Venezuela (VEN), Malaysia (MYS), Afghanistan (AFG) and Thailand (THA). Each dot represents an amplicon. Use case-groups were defined as 169 amplicons for geographic differentiation, 32 amplicons for drug resistance surveillance (DRS), and the four amplicons conforming PvCRiSP.
(TIFF)

S13 Fig Panel discrimination power. The figure on the left shows the probability that two random samples differ from each other in at least one amplicon (x-axis) across global *P. vivax* populations (y-axis) using PvGTSeq (blue) and PvCRiSP (red). Figure on the right shows the distribution of identity by state within each population measured from whole genome sequencing data.
(TIFF)

S14 Fig Scatter plots depicting Tajima’s D values in each amplicon included in the PvGTSeq panel. Shapes of the dots represent whether the amplicon is used for geographic differentiation (triangles) or for drug resistance surveillance (circles) and dark gray color indicates where the amplicon is under strong selection signal (Tajima’s D > 2 or <-2). The analyzed countries includes: Ethiopia (ETH), Cambodia (KHM), Vietnam (VNM), Brazil (BRA), Colombia (COL), Guyana (GUY), Honduras (HND), Peru (PER), Venezuela (VEN), Afghanistan (AFG) and Thailand (THA), and numbers within parenthesis indicates the number of non-clonal haplotypes used in the analysis.
(TIFF)

S15 Fig Principal coordinate analysis between Africa (AF) and Western Asia (WAS) in panel A, and between Eastern South-East Asia (ESEA), Maritime South-East Asia (MSEA), Oceania (OCE), and Western South-East Asia (WSEA) in panel B. (TIFF)

S16 Fig Network graph showing genetic relationships (IBS) among samples from Colombia, Honduras, Mexico and Panama. Each node represents a monoclonal sample, with edges indicating genetic relationships exceeding 0.6 IBS. Panels B, C and D share the same color scheme.
(TIFF)

S17 Fig Principal coordinate analysis of samples from Guyana generated through PvGTSeq (light blue dots) and WGS (blue dots). (TIFF)

S1 Text PvGTSeq protocol. (DOCX)

S2 Text PvCRiSP protocol. (DOCX)

S1 Table List of amplicons included in the PvGTSeq and PvCRisP panel. (XLSX)

S2 Table Prevalence by country of haplotypes of genes that carry mutations associated with antimalarial resistance. (XLSX)

S3 Table LOD, amplification rate and read depth of each amplicon in PvGTSeq by country. (XLSX)

S4 Table LOD of PvGTSeq and PvCRiSP by country. (XLSX)

S5 Table LOD, amplification rate and read depth of each amplicon in PvCRiSP by country. (XLSX)

S6 Table Cost breakdown of PvGTSeq and PvCRiSP. (XLSX)
