# Supplementary figures and images for: PvGTSeq and PvCRiSP: Two amplicon-based targeted sequencing panels for Plasmodium vivax
Source: PLoS Negl Trop Dis. 2026 May 14;20(5):e0013663. doi: 10.1371/journal.pntd.0013663 (PMC13189417; doi:10.1371/journal.pntd.0013663)

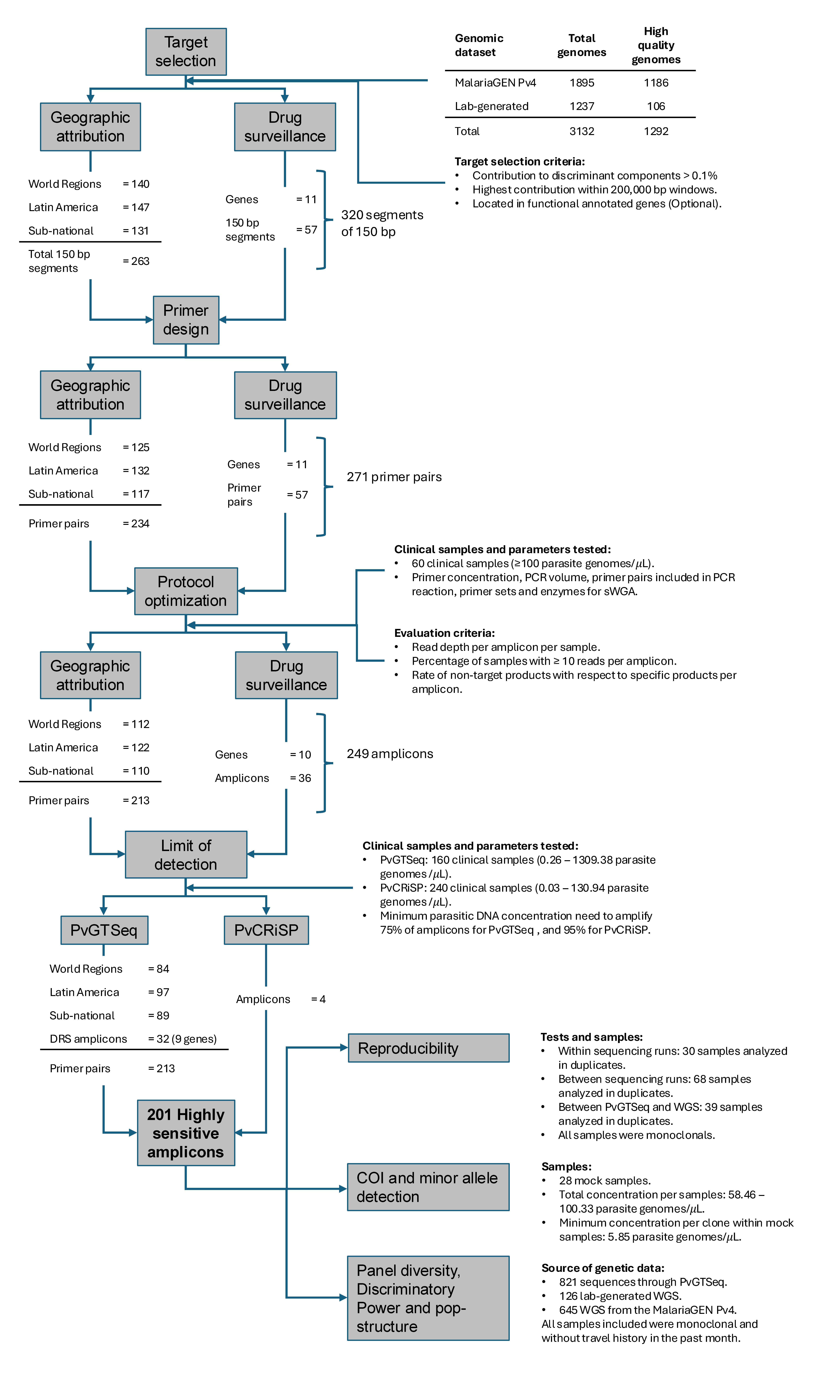

Supplement: S1 Fig — Each stage details the number of amplicons (segments or primer pairs) selected, the samples or genomic data used for the analysis, and the evaluation criteria applied. (TIFF) [file pntd.0013663.s001.tiff]

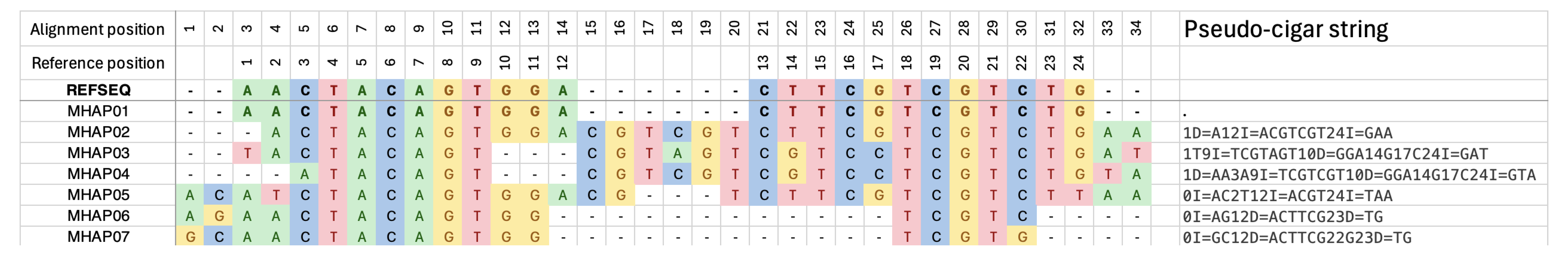

Supplement: S2 Fig — In our analysis pipeline, after dada2 denoising of sequencing errors, all microhaplotypes are aligned to the reference sequence using MUSCLE (Multiple Sequence Comparison by Log-Expectation). Polymorphisms are summarized in Pseudo-cigar format following these rules: 1) All variants are annotated in ascending order. 2) For SNVs, we annotated the reference position followed by the substitute nucleotide. 3) For deletions, we annotated the starting position, “D=”, then all deleted nucleotides (e.g., 23D = TG). 4) For insertions, we annotated the position before insertion, “I=”, then the nucleotide at that position followed by inserted nucleotides. 5) If the nucleotide before an insertion is an SNV, we include this SNV within the insertion notation to avoid position duplication (see MHAP05). 6) When a deletion is followed by an insertion, the insertion is annotated first to maintain ascending order (see MHAP03 and MHAP04). 7) For insertions before position 1, we use position 0 and only annotate inserted nucleotides (see MHAP05-MHAP07). 8) If both reference and microhaplotype have deletions, but the microhaplotype deletion extends beyond the reference deletion, we consider it a single deletion (see MHAP06 and MHAP07). 9) If both, the reference and the microhaplotype, have deletions, but the reference deletion extends beyond the microhaplotype deletion, we consider it a single insertion (see MHAP05). 10) Microhaplotypes that are identical to the reference sequence are annotated using a period symbol “.” (see MHAP01). (TIFF) [file pntd.0013663.s002.tiff]

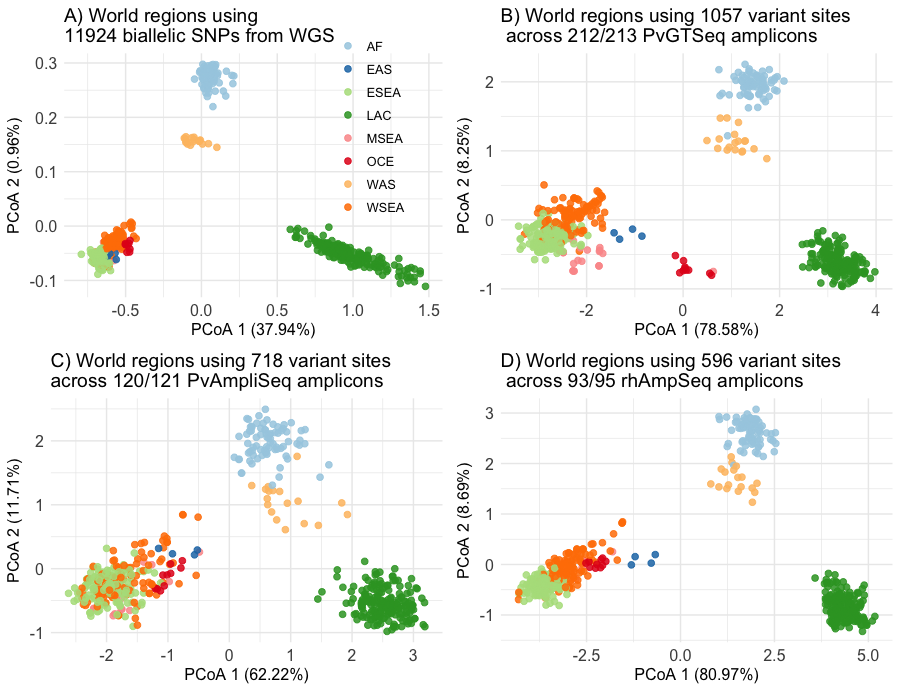

Supplement: S3 Fig — The analysis was done using genomic information from MalariaGEN Pv4, Brazilian genomes available from the European Nucleotide Archive (PRJEB56411, PRJEB44419, PRJEB36199) and 112 genomes from Colombia, Guyana, Honduras, Panama, Peru and Venezuela generated in our group. The analysis at world regions scale includes: Africa (AF), East Asia (EAS), Eastern Southeast Asia (ESEA), Latin America and Caribbean (LAC), Maritime South-East Asia (MSEA), Oceania (OCE), Western Asia (WAS), and Western South-East Asia (WSEA). (TIFF) [file pntd.0013663.s003.tiff]

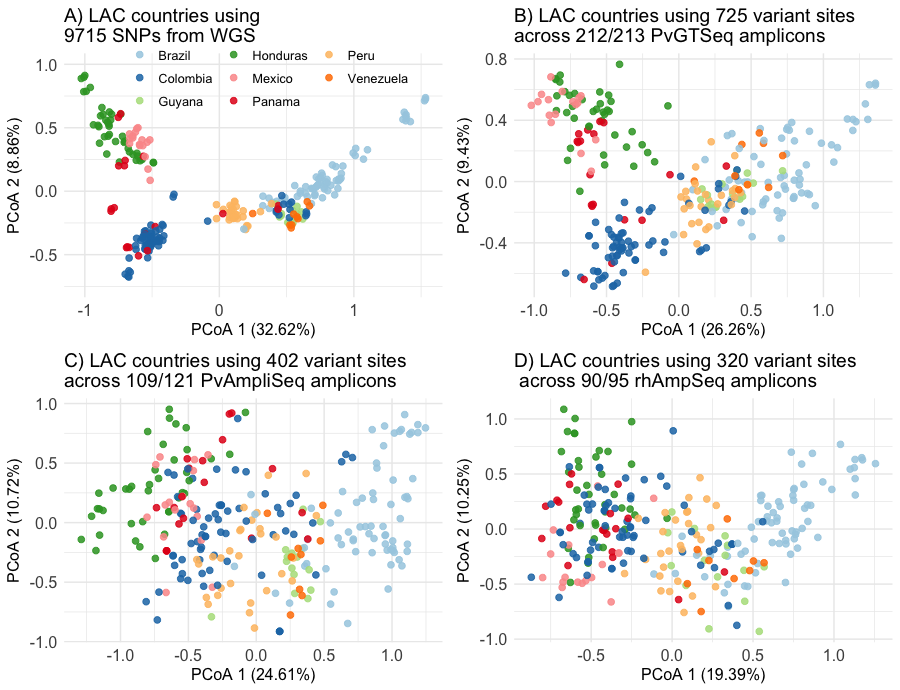

Supplement: S4 Fig — The analysis was done using genomic information from MalariaGEN Pv4, Brazilian genomes available from the European Nucleotide Archive (PRJEB56411, PRJEB44419, PRJEB36199), and 112 genomes from Colombia, Guyana, Honduras, Panama, Peru and Venezuela. (TIFF) [file pntd.0013663.s004.tiff]

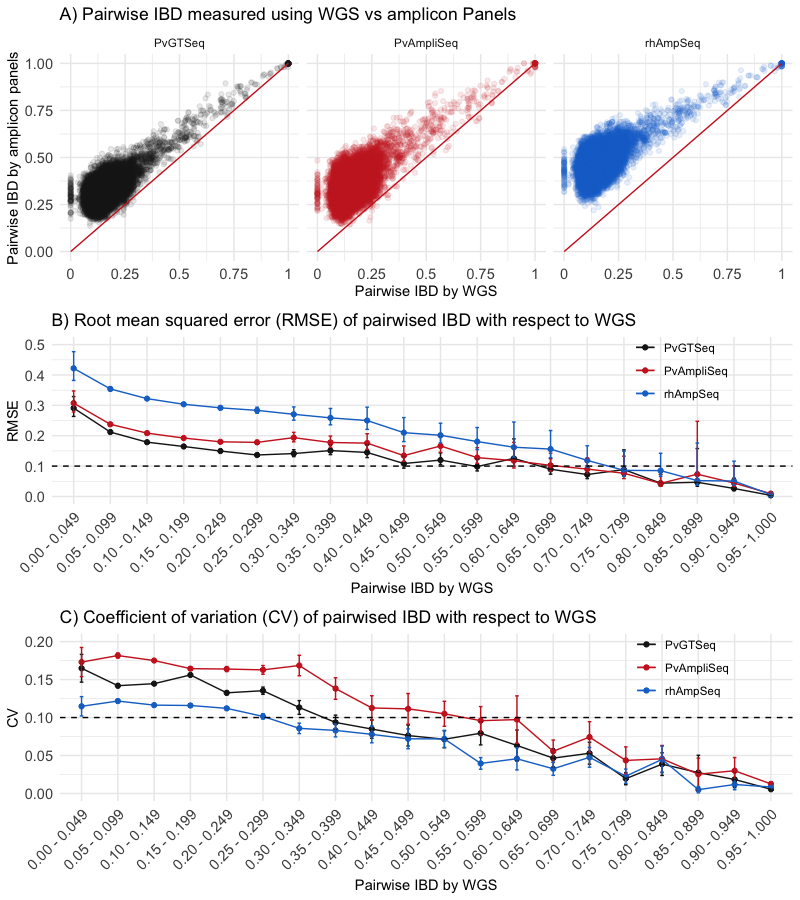

Supplement: S5 Fig — A) Scatter plot of IBD estimates for pairs of samples using WGS (x-axis) and the amplicon panels (y-axis). Each point represents a pairwise comparison, and the diagonal line indicates the expected value when IBD estimates from WGS and the panels are the same. B) Root mean squared error of IBD estimates from the amplicon panels relative to WGS across different IBD ranges. Confidence intervals were constructed assuming a chi-squared distribution. C) Coefficient of variation of IBD estimates from the amplicon panels relative to WGS across different IBD ranges. Confidence intervals were constructed assuming a T distribution. (TIFF) [file pntd.0013663.s005.tiff]

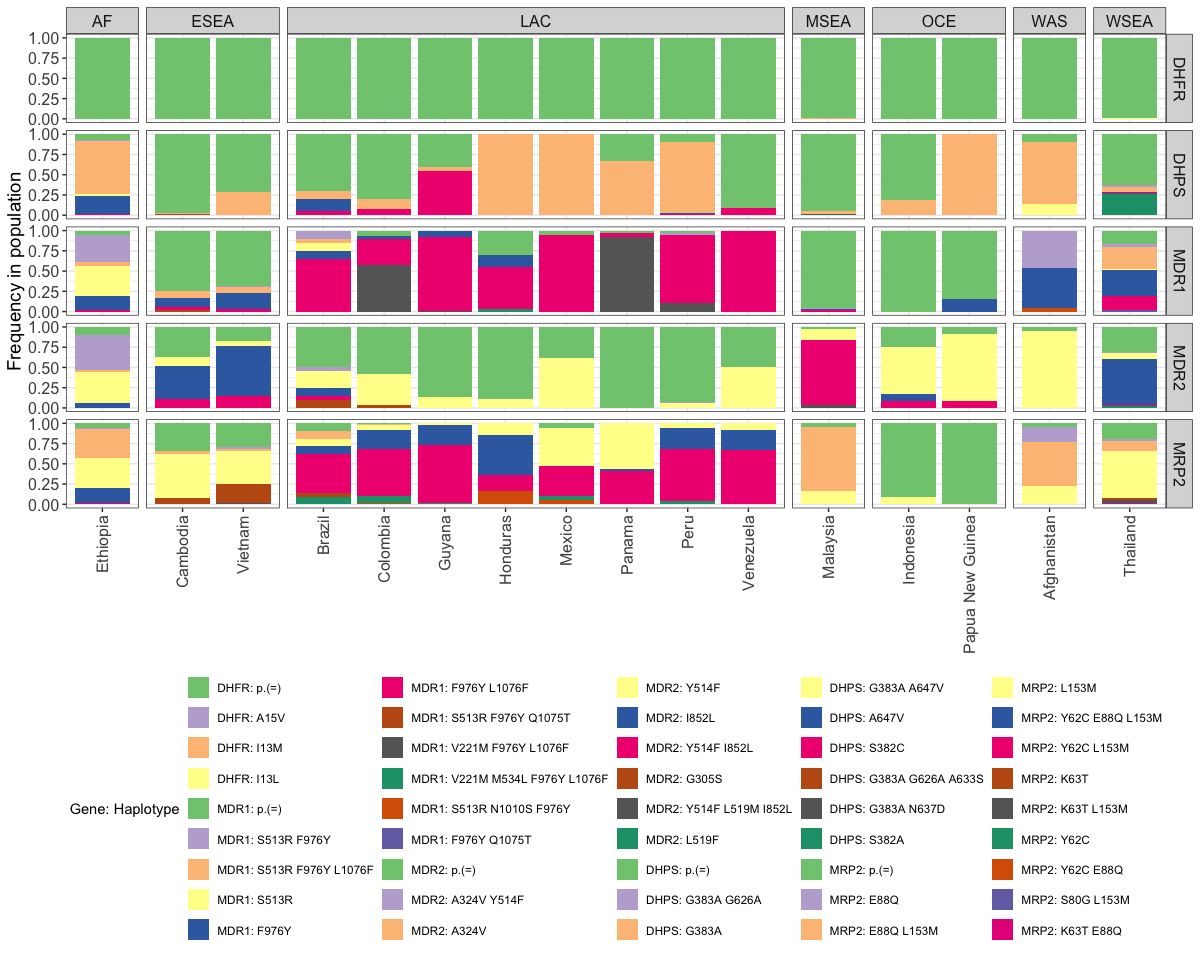

Supplement: S6 Fig — (TIFF) [file pntd.0013663.s006.tiff]

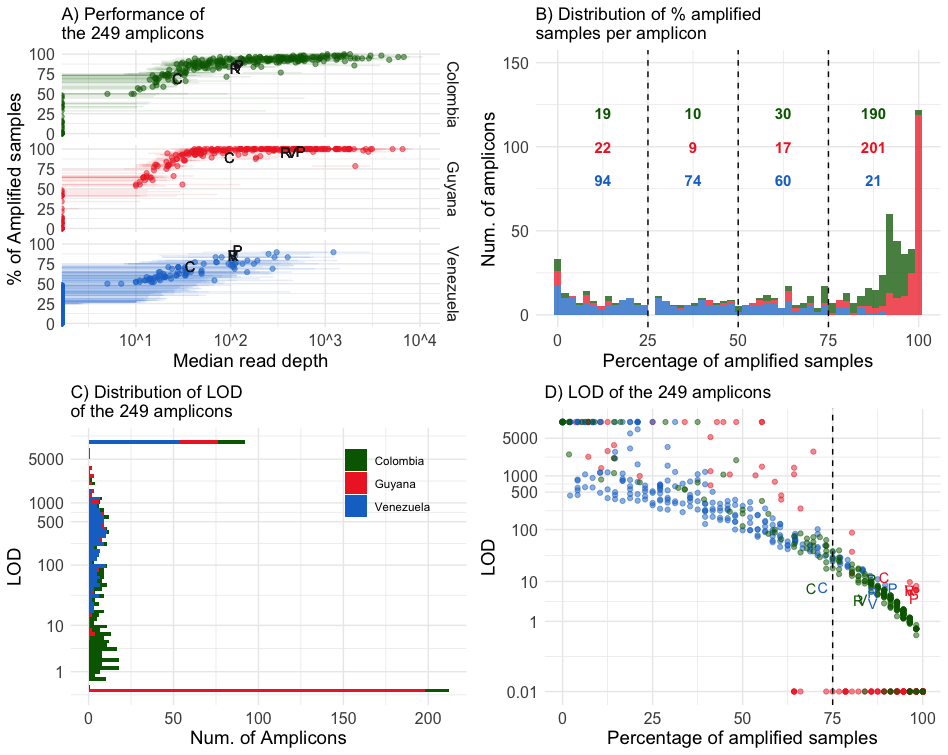

Supplement: S7 Fig — Dots represent each of the 249 amplicons in PvGTSeq while the letters represent the 4 amplicons in PvCRiSP (CG2_releated, RIPR, VSP11, and PIGM). B) Distribution of amplification rate of the amplicons in PvGTSeq and PvCRiSP in the three countries, as indicated by color. Numbers in panel B indicate the number of amplicons in each population that are in the 1st, 2nd, 3rd, and 4th quantile. C) Distribution of limit of detection (LOD) of each individual amplicon in PvGTSeq and PvCRiSP. D) Scatter plot of amplification rate (x-axis) and LOD (y-axis) of each individual amplicon (dots or letters) in three populations. (TIFF) [file pntd.0013663.s007.tiff]

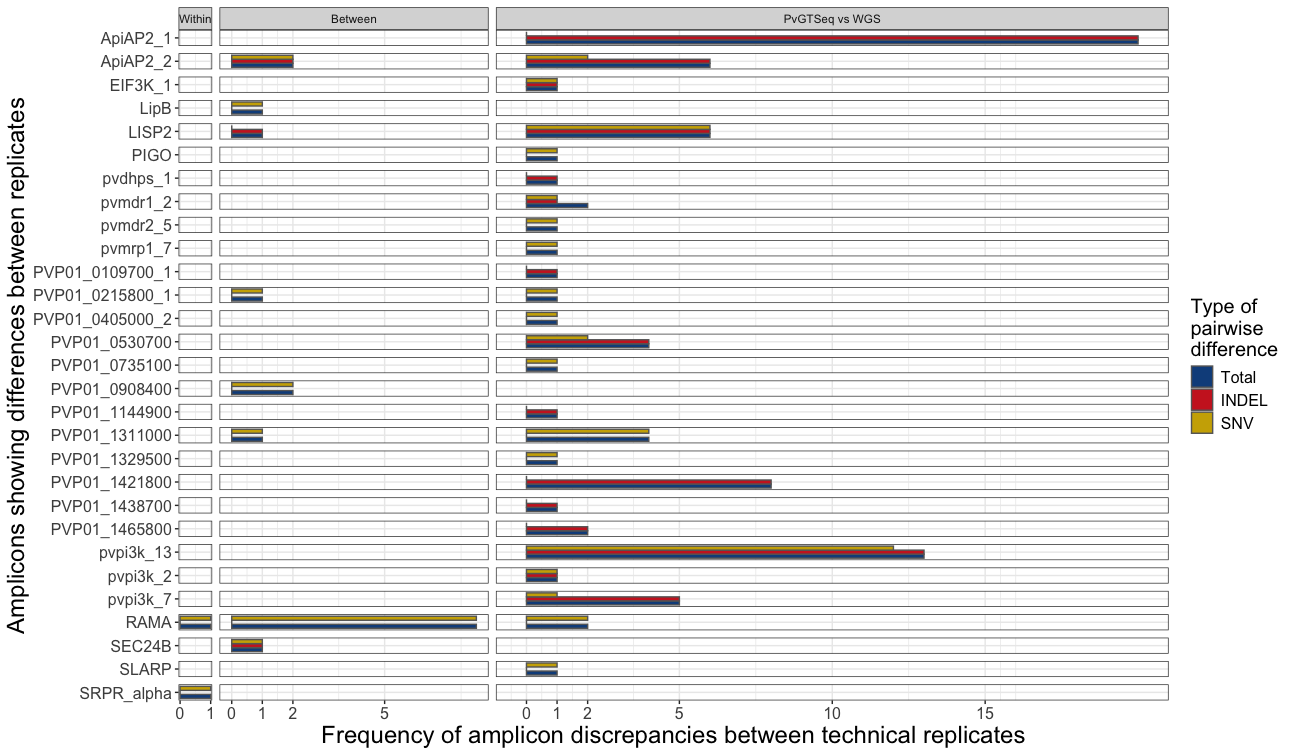

Supplement: S8 Fig — X-axis shows the number of times an amplicon (y-axis) showed a discrepancy between technical replicates in any of the 3 experiments (Vertical panels): Within and between sequencing runs of PvGTSeq, and between PvGTSeq and WGS. Colors indicate if the discrepancy in the amplicon was due to a single nucleotide variant (SNV, in gold), an insertion or deletion (INDEL, in red) or any of both (in blue). (TIFF) [file pntd.0013663.s008.tiff]

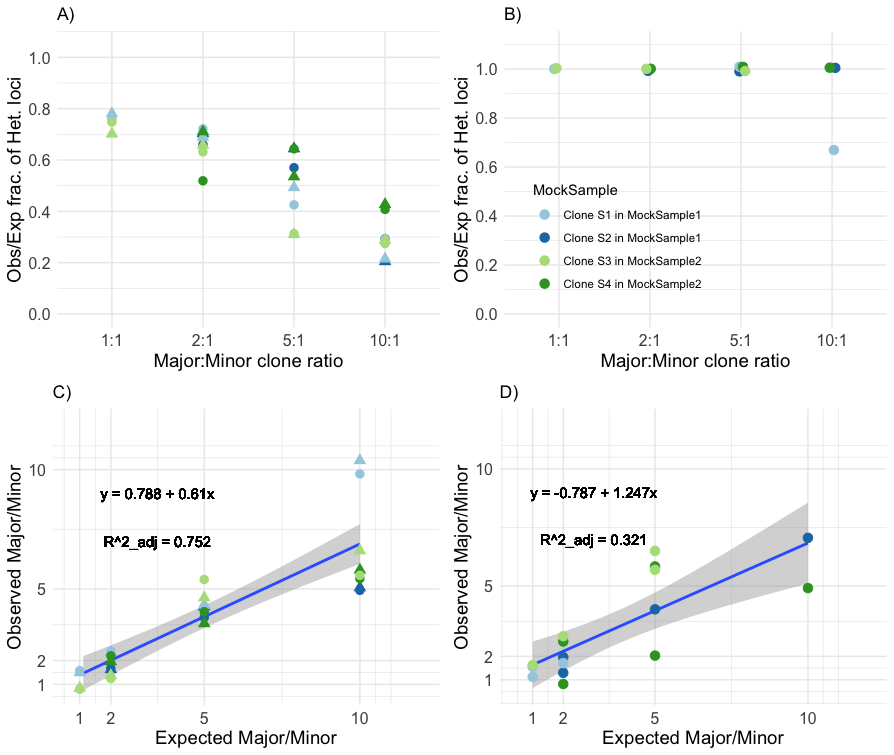

Supplement: S9 Fig — The bottom panels illustrate the correlation between the read depth ratio of major and minor clones for PvGTSeq (C) and PvCRiSP (D) compared to their DNA concentration ratios. MS1 and MS2 represent the two biological replicates, and shapes of the dots (circles and triangles) represent the 2 technical replicates. (TIFF) [file pntd.0013663.s009.tiff]

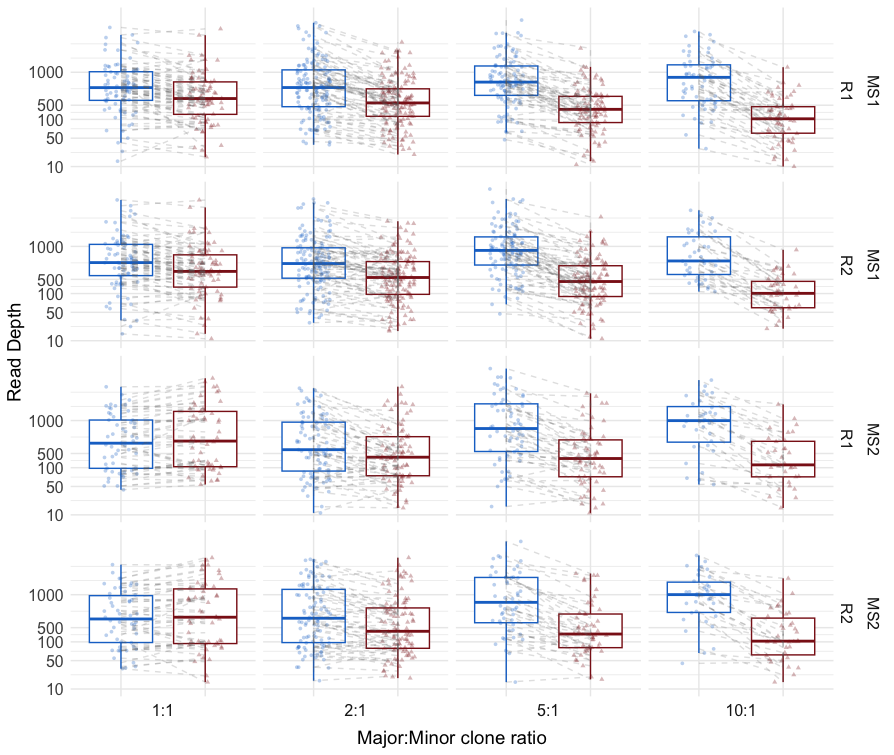

Supplement: S10 Fig — Each dot represents a private microhaplotype from major (blue) and minor (red) clones using the 201 amplicons from PvGTSeq. Horizontal panels represent each of the two combinations of mock samples (MS1 and MS2) and their technical replicates (R1 and R2), while the vertical panels represent the different ratios at which mock samples were generated. (TIFF) [file pntd.0013663.s010.tiff]

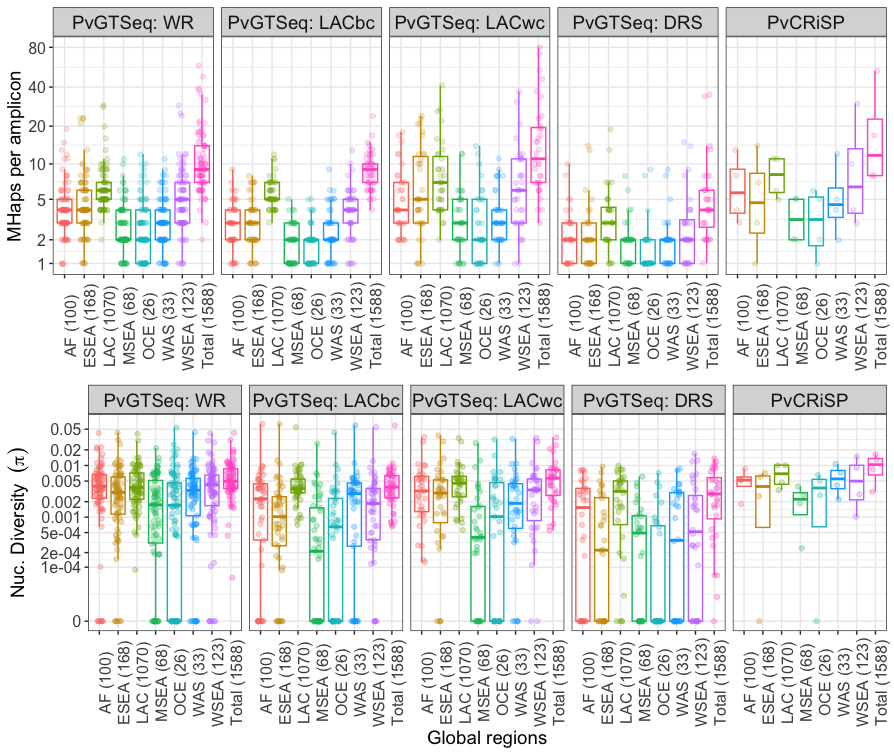

Supplement: S11 Fig — Numbers within parenthesis indicate the number of monoclonal clinical samples. Each dot represents an amplicon. Each dot represents an amplicon. Use case groups were defined as 81 amplicons for geographic differentiation between world regions (WR), 49 amplicons for geographic differentiation between countries in LAC (LACbc), 35 amplicons for geographic differentiation within countries in LAC (LACwc), 32 amplicons for drug resistance surveillance (DRS), and the four amplicons comprising PvCRiSP. Because there are amplicons for geographic differentiation that belong to multiple groups, to avoid duplication we assigned the amplicon to the highest geographical scale. (TIFF) [file pntd.0013663.s011.tiff]

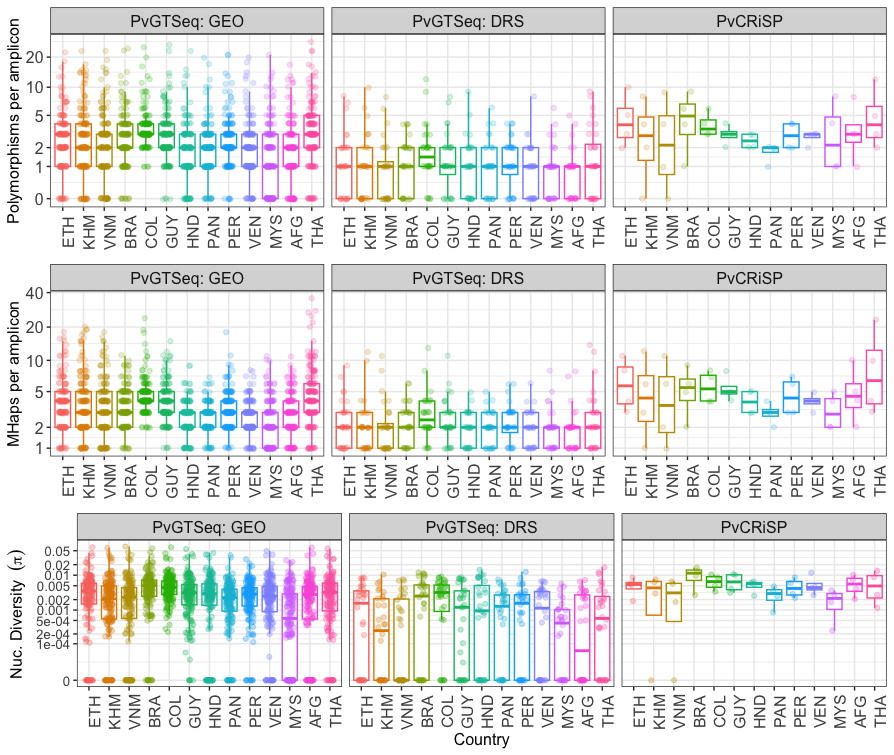

Supplement: S12 Fig — Each dot represents an amplicon. Use case-groups were defined as 169 amplicons for geographic differentiation, 32 amplicons for drug resistance surveillance (DRS), and the four amplicons conforming PvCRiSP. (TIFF) [file pntd.0013663.s012.tiff]

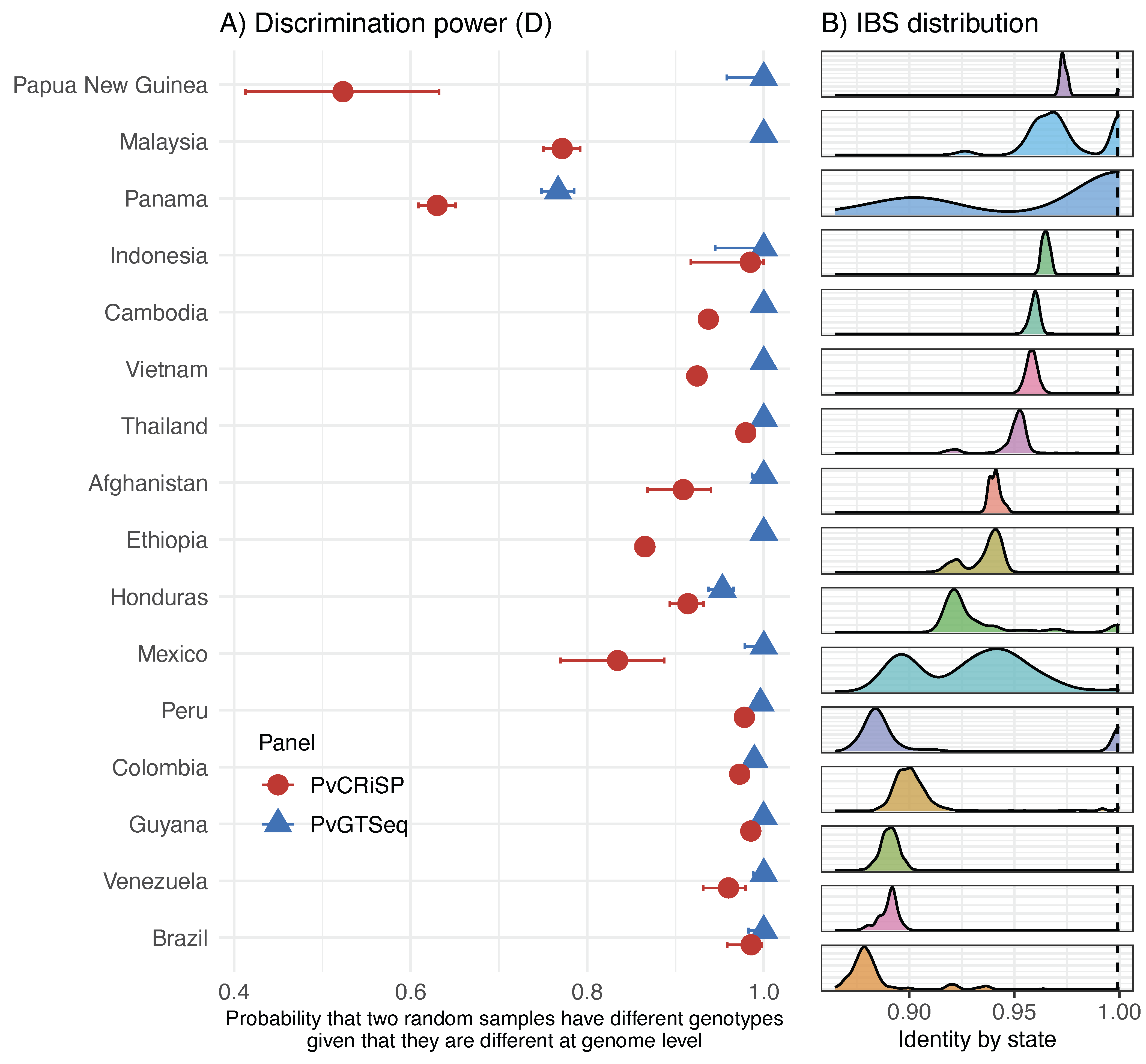

Supplement: S13 Fig — The figure on the left shows the probability that two random samples differ from each other in at least one amplicon (x-axis) across global P. vivax populations (y-axis) using PvGTSeq (blue) and PvCRiSP (red). Figure on the right shows the distribution of identity by state within each population measured from whole genome sequencing data. (TIFF) [file pntd.0013663.s013.tiff]

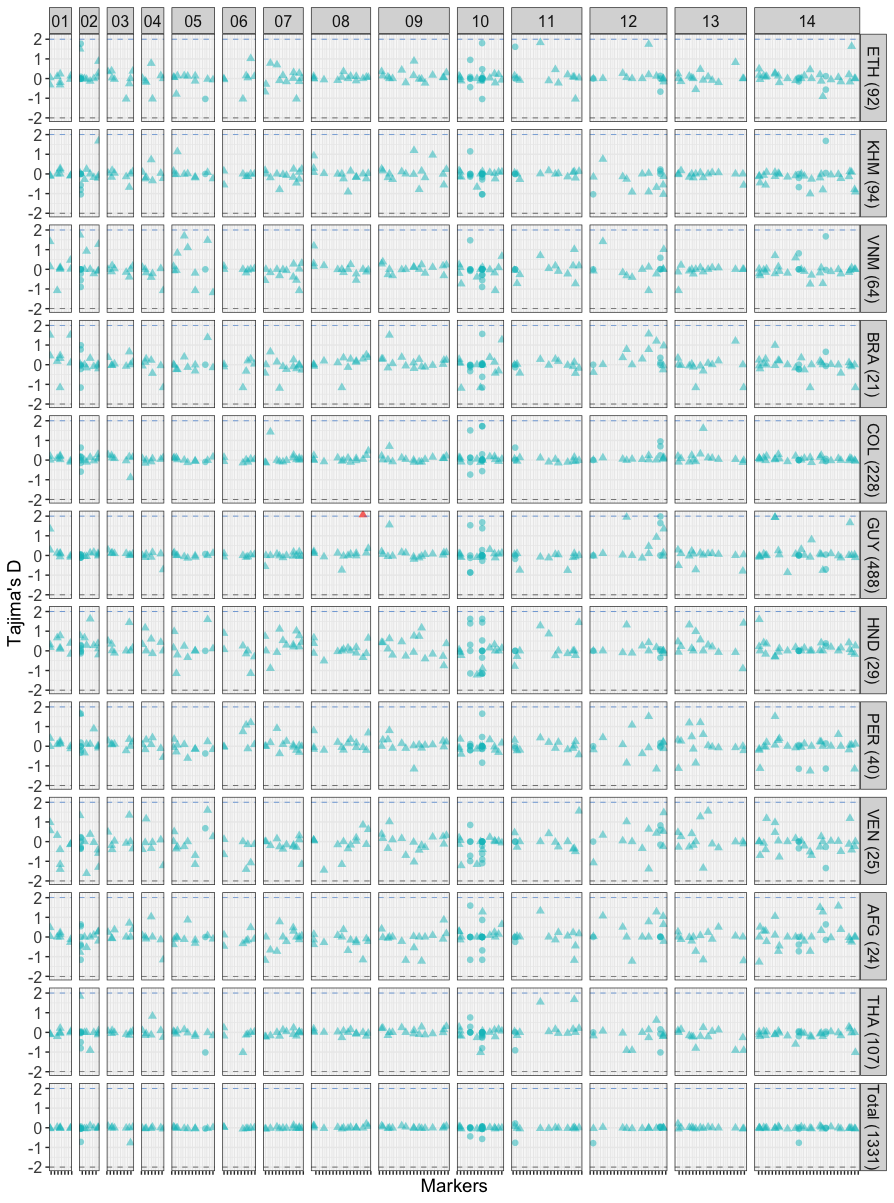

Supplement: S14 Fig — Shapes of the dots represent whether the amplicon is used for geographic differentiation (triangles) or for drug resistance surveillance (circles) and dark gray color indicates where the amplicon is under strong selection signal (Tajima’s D > 2 or <-2). The analyzed countries includes: Ethiopia (ETH), Cambodia (KHM), Vietnam (VNM), Brazil (BRA), Colombia (COL), Guyana (GUY), Honduras (HND), Peru (PER), Venezuela (VEN), Afghanistan (AFG) and Thailand (THA), and numbers within parenthesis indicates the number of non-clonal haplotypes used in the analysis. (TIFF) [file pntd.0013663.s014.tiff]

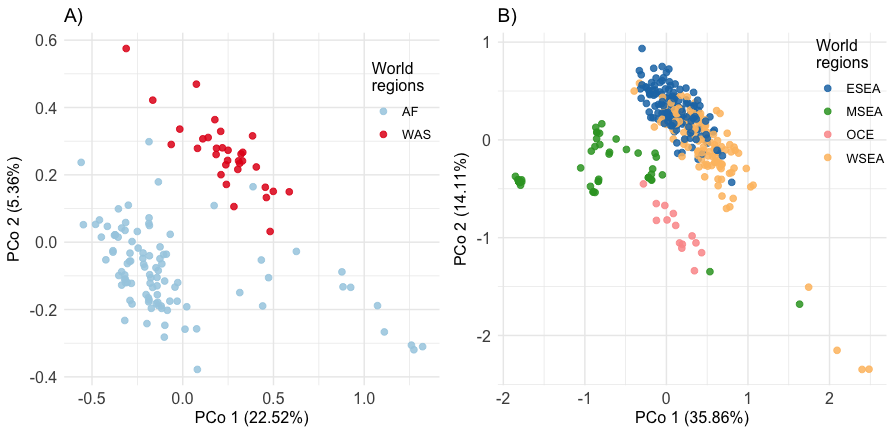

Supplement: S15 Fig — (TIFF) [file pntd.0013663.s015.tiff]

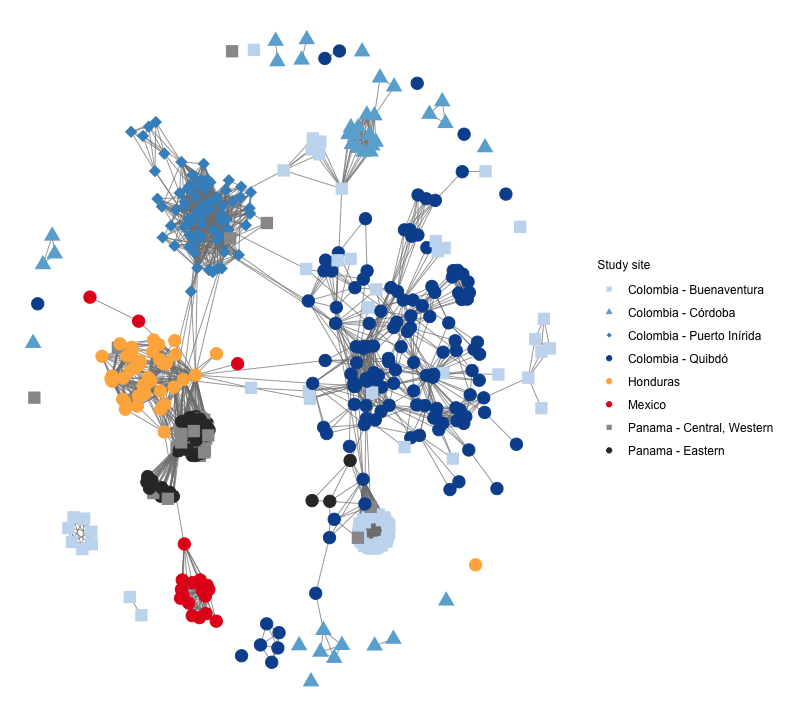

Supplement: S16 Fig — Each node represents a monoclonal sample, with edges indicating genetic relationships exceeding 0.6 IBS. Panels B, C and D share the same color scheme. (TIFF) [file pntd.0013663.s016.tiff]

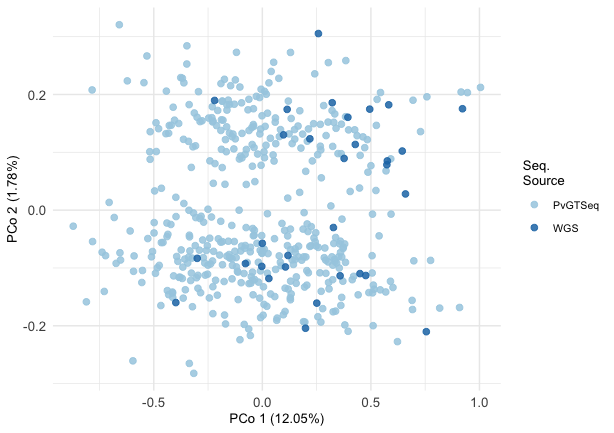

Supplement: S17 Fig — (TIFF) [file pntd.0013663.s017.tiff]
